# PC3/Tis21/BTG2 and BTG1 genes: regulators of the cell cycle and neurogenesis, as well as tumor suppressors in malignant brain tumors

**DOI:** 10.3389/fcell.2026.1775035

**Published:** 2026-04-14

**Authors:** Manuela Ceccarelli, Laura Micheli, Giorgio D'Andrea, Felice Tirone

**Affiliations:** 1 Onco-Hematology, Cell Therapy, Gene Therapies and Hemopoietic Transplant, Bambino Gesù Children’s Hospital, IRCCS, Rome, Italy; 2 Institute of Biochemistry and Cell Biology, National Research Council, Monterotondo Scalo, Rome, Italy

**Keywords:** apoptosis, BTG1, BTG2, cell cycle, glioma, medulloblastoma, neurogenesis

## Abstract

*PC3/Tis21/BTG2* and *BTG1*, prototype members of the *BTG*/*Tob* family, are antiproliferative transcriptional cofactors discovered 35 years ago as genes induced by nerve growth factor and phorbol 12-myristate 13-acetate or associated with lymphocytic leukemia. They are today known to serve as developmental regulators in several tissues, including neural cells. Biological functions such as cell division, transcriptional control, DNA repair, and mRNA stability, have been linked to their protein products. We will focus in this review on the effects of *PC3*/*Tis21*/*BTG2* and *BTG1* on brain tumorigenesis and neural development, and on cell cycle and apoptosis. In fact, these genes act as tumor suppressors, and their ability to control tumorigenesis in medulloblastoma and glioma is intrinsically linked to their ability to control the differentiation and proliferation of neural stem and progenitor cells during neurogenesis. Chief function of *PC3*/*Tis21*/*BTG2* during pre/postnatal and adult neurogenesis is its requirement for the differentiation and migration of neural progenitor cells, in adult hippocampus and subventricular zone–which are the main neurogenic niches where adult neurogenesis occurs–as well as in postnatal cerebellum. Moreover, *PC3*/*Tis21*/*BTG2* inhibits medulloblastoma onset by promoting the migration and differentiation of cerebellar precursor cells outside the external granular layer, i.e., the proliferative epithelium of the cerebellum, thus diminishing their susceptibility to oncogenic transformation under the influence of Sonic Hedgehog. *BTG1*, by contrast, primarily functions in neurogenesis to inhibit the proliferation of neural stem and progenitor cells, thereby ensuring the preservation of the cell pool and maintaining the quiescence of medulloblastoma cancer stem cells–known for their persistence against treatments and involvement in tumor relapses–thus preventing their entry in cycle. Furthermore, in glioma, *PC3*/*Tis21*/*BTG2* enhances apoptosis rates while simultaneously decreasing the migration and invasion of cancerous cells, and lowering the levels of *cyclin D1*. Similarly, *BTG1* contributes to the growth arrest of glioma cells through the regulation of *cyclin D1* and *p21* expression. *PC3/Tis21*/*BTG2* and BTG1 bind and regulate multiple genes, including *Id3*, *cyclin D1*, *PRMT1* and the chemokine *Cxcl3*. These interactions underscore the potential of these cofactors in controlling neurogenesis and tumorigenesis through multiple molecular pathways.

## 1 Introduction

*PC3*/*Tis21*/*BTG2* and *BTG1* are transcriptional cofactors negative regulators of cell cycle, and are involved in several biological processes including neural differentiation and maintenance of neural stem cells, apoptosis, myoblast proliferation, vertebral patterning, development of hematopoietic progenitor cells and response to genotoxic stress. Moreover, *PC3*/*Tis21*/*BTG2* and *BTG1* play an important role as tumor suppressors. After an introduction on their isolation, structure and the signaling pathways they activate, we will focus on their functional effects on brain tumorigenesis and neural development, cell cycle and apoptosis, which are related to the tumor suppressor activity of *PC3*/*Tis21*/*BTG2* and *BTG1* in the brain.

## 2 Isolation, structure and transcriptional regulation by *PC3*/*Tis21*/*BTG2* and *BTG1*

The murine orthologue of *BTG2*, *PC3*, was first identified in the rat pheochromocytoma PC12 cells, a neural crest-derived cell line, during the early stages of sympathetic neuron differentiation activated by nerve growth factor (NGF; Bradbury et al., 1991). Meanwhile, NIH3T3 mice fibroblasts were found to express the mouse orthologue *Tis21* as a phorbol ester-induced gene (Fletcher et al., 1991); the human orthologue of *PC3*, i.e., *BTG2*, was later identified as a p53-inducible gene (Rouault et al., 1996). The same year of the discovery of the *PC3*/*Tis21* gene was identified *BTG1* as a translocation partner of the *c-Myc* gene in a case of B-cell chronic lymphocytic leukemia (Rimokh et al., 1991).

*BTG1* and *BTG2* are the first genes isolated of a family of six related genes with antiproliferative activity, *BTG1*, *BTG2*/*PC3*/*Tis21*, *BTG3*/*ANA*, *BTG4*/*PC3B*, *Tob1*/*Tob*, and *Tob2* (Tirone, 2001; Winkler, 2010). These proteins share two conserved regions, Box A or GR and Box B. The removal of Box GR/Box A in *PC3* abolishes its ability to inhibit cell proliferation and suppress *cyclin D1* expression (Guardavaccaro et al., 2000; see below). A third box, Box C, is present only in *BTG1* and *BTG2*, which are the genes sharing the greater homology within the family. All these conserved domains are involved in the control of proliferation. For analysis of homologies and phylogenetic relationships between protein sequences of the BTG/Tob family see Tirone (2001) and Winkler (2010).

PC3/Tis21/BTG2 (as we also refer to BTG2) is a transcriptional regulator that controls transcription by binding to the promoters of multiple genes, such as *cyclin D1* (Farioli-Vecchioli et al., 2007), *RARβ* (Passeri et al., 2006), *Id3* (Farioli-Vecchioli et al., 2009), and *Cxcl3* (Farioli-Vecchioli et al., 2012a).

PC3/Tis21/BTG2 exerts its function as part of protein complexes that incorporate various transcriptional elements like Caf1/CNOT8 (Rouault et al., 1998; Prévôt et al., 2001), the transcription factor HoxB9 (Prévôt et al., 2000), which is positively modulated by BTG2 and BTG1 and is responsible for pattern formation during development. In addition, PC3/Tis21/BTG2 interacts with histone modifying factors, such as the methyltransferase PRMT1 (Lin et al., 1996; Passeri et al., 2006) and the histone deacetylases HDAC4 or HDAC1 (Passeri et al., 2006; Farioli-Vecchioli et al., 2007; Micheli et al., 2017b). Moreover, two LXXLL motifs present in both BTG2 and BTG1 proteins modulate the interaction with several nuclear receptors, e.g., the all-trans retinoic acid (RA) receptor, the triiodothyronine (T3) receptor, the estrogen receptor α (ERα) and the androgen receptor (Busson et al., 2005; Prevot et al., 2001; Hu et al., 2011). PRMT1 catalyzes arginine methylation of proteins, including histones, and binds the box C of BTG2 and BTG1 (Lin et al., 1996). A primary role of PRMT1-mediated arginine methylation is to facilitate transcription, and BTG2 increases PRMT1 participation in the RA protein complex on the *RARβ* promoter, with the result that PRMT1 enhances RA transcription and RA-induced differentiation of myeloid leukemia cells (Passeri et al., 2006). Similarly, it has been shown that PRMT1 binds BTG1 (Lin et al., 1996) and that BTG1-PRMT1 complexes activate glucocorticoid receptor signaling in leukemic cells (van Galen et al., 2010). Moreover, BTG1 improved insulin sensitivity by promoting *c-Jun* expression through stimulation of c-Jun and RA receptor activities (Xiao et al., 2016). Interestingly, PC3/Tis21/BTG2 binds the promoter of the anti-differentiative gene *Id3*, and inhibits its transcription (Farioli-Vecchioli et al., 2009). Since Id3 lacks a DNA-binding domain but possesses a helix-loop-helix (HLH) dimerization domain, it sequesters E proteins and prevents them from attaching to proneural basic HLH transcription factors, which in turn causes the transcription of those proteins to be inactivated, including *NeuroD1*, required for the maturation of hippocampal granule progenitor cells in differentiated neurons. As a result, PC3/Tis21/BTG2 promotes neural differentiation through negative modulation of *Id3* transcription (Farioli-Vecchioli et al., 2009). Moreover, PC3/Tis21/BTG2 inhibits *cyclin D1* expression and cell cycle by binding to *cyclin D1* promoter and to HDAC1 and HDAC4; deletion of *HDAC1* and *HDAC4* impairs the ability of PC3/Tis21/BTG2 to inhibit *cyclin D1* expression (Farioli-Vecchioli et al., 2007; Micheli et al., 2017b). This indicates that *PC3*/*Tis21*/*BTG2* inhibits cell proliferation in a way dependent on the presence of HDACs, in neural cells and in fibroblasts. Furthermore, PC3/Tis21/BTG2 binds to the promoter of the chemokine *Cxcl3*, stimulating its transcription (Farioli-Vecchioli et al., 2012a); in turn, Cxcl3 promotes the migration of cerebellar precursors outside the proliferative epithelium (external granular layer) to the internal cerebellar layers, where cerebellar precursors differentiate, withdrawing from the neoplastic program (Ceccarelli et al., 2016; 2025; see section on medulloblastoma).

## 3 Pathways controlling the expression of *PC3*/*Tis21*/*BTG2* and *BTG1*

*PC3*/*Tis21*/*BTG2* transcription is inducible by a variety of growth factors. These include NGF, fibroblast growth factor (FGF), epidermal growth factor (EGF) and interleukin 6 (IL-6) (Bradbury et al., 1991), which is an important mediator of inflammation. The induction of *PC3*/*Tis21*/*BTG2* by growth factors was confirmed by other studies (Altin et al., 1991). *PC3*/*Tis21*/*BTG2* transcription is activated also by cell depolarization in neural cells and in the brain; it can also be triggered by cyclic AMP, a messenger responsible for the activation of several proteins, such as protein kinase A, which regulates by phosphorylation several cell functions (Bradbury et al., 1991). Moreover, *BTG2* is induced by DNA damage through genotoxic agents (ionizing radiation, UV, adriamycin) in consequence of p53 induction (Rouault et al., 1996). *BTG2* expression is also activated by the tumor promoter tetradecanoylphorbol acetate (TPA), which activates protein kinase C (Fletcher et al., 1991). Conversely, in breast cancer, estrogen suppresses *BTG2* expression, an effect mediated by estrogen’s interaction with the estrogen receptor, particularly ERα (Karmakar et al., 2009). Moreover, breast cancer proliferation is arrested by retinoic acid through its ability to induce several genes, chiefly *BTG2* (Donato et al., 2007).

As for *BTG1*, its expression is induced by DNA damage but in a p53-independent way (Cortes et al., 2000). It is induced also by glucorticoids (van Galen et al., 2010), by transforming growth factor β (TGF-β) and angiogenic growth factors (Iwai et al., 2004), as well as by T3 and cyclic AMP (Marchal et al., 1995).

Thus, *PC3*/*Tis21*/*BTG2* and *BTG1* are regulated by several growth factors and hormone receptors pathways.

## 4 Posttranscriptional regulation

It has been demonstrated that BTG2 promotes mRNA poly(A) tail shortening by binding the CAF1 deadenylase (part of the CCR4–NOT complex, which bears the main catalytic activity for cytoplasmic deadenylation). Through this interaction BTG2 significantly accelerates poly(A) tail removal across transcripts and promotes mRNA decay in mammalian cells (Mauxion et al., 2008). This deadenylation effect by BTG2 is non-specific, as it occurs indiscriminately on all mRNAs (e.g., β-globin or cyclin D1).

A first work indicated that BTG2’s antiproliferative activity depends on CAF1a/CAF1b interaction (Doidge et al., 2012). Further studies have shown that BTG2 bridges the first RRM domain of the poly(A)-binding protein PABPC1 and CAF1. Through this interaction, BTG2 stimulates the deadenylase activity of CAF1 *in vitro*. The formation of this tripartite complex is essential for the ability of *BTG2* to suppress cell proliferation (Stupfler et al., 2016). This finding suggests that the antiproliferative property of *BTG2* observed in U2OS cells depends on mRNA deadenylation. However, it remains unclear whether this deadenylation-dependent antiproliferative activity is specific or merely reflects the global mRNA deadenylation activity exerted by BTG2 (Stupfler et al., 2016).

## 5 Regulation of biological processes by *PC3*/*Tis21*/*BTG2* and *BTG1*

### 5.1 Negative regulation of cell cycle by *PC3*/*Tis21*/*BTG2*

The first evidence that *PC3*/*Tis21*/*BTG2* inhibits the cell cycle came from experiments of overexpression of *PC3* in neural cells (PC12 cells) and cell cycle synchronization. The authors showed that *PC3*/*Tis21*/*BTG2* impairs the transition from G1 to S phase, with decrease of proliferation and colony formation, as well as hypophosphorylation of pRb, indicative of cell cycle arrest (Montagnoli et al., 1996). Further studies indicated that *PC3* impairs the G1-S transition by inhibiting pRb function in consequence of a downregulation of *cyclin D1* levels (Guardavaccaro et al., 2000) in mouse fibroblasts. The suppression of *cyclin D1* levels was shown to depend on the interaction of PC3/Tis21/BTG2 with the histone deacetylases HDAC1, HDAC4 and HDAC9. This study provided mechanistic detail by identifying specific HDAC partners, although it was conducted mainly in biochemical and overexpression systems (Micheli et al., 2017b). Notably, the role of *cyclin D1* in the inhibition of proliferation by *PC3*/*Tis21*/*BTG2* is selective, as this is the only cyclin able to significantly rescue the G1 arrest exerted by *PC3*/*Tis21*/*BTG2* (Guardavaccaro et al., 2000). Examples of inhibition of the G1-S phase by *PC3*/*Tis21*/*BTG2* in non-neural cells are in mouse embryo fibroblasts and embryonic stem cells (Rouault et al., 1996; Boiko et al., 2006), in breast (Kawakubo et al., 2004) and prostate cancer cells (Ficazzola et al., 2001) or in granulosa cell of the ovary (Li et al., 2009). These studies collectively show broad reproducibility across tissues, although most use single cell lines and rely on overexpression or knockdown approaches.

This G1-S phase arrest is exerted by inhibition of the expression of *cyclin D1* and of the activity of Cdk4/cyclin D1 complexes on pRb (Guardavaccaro et al., 2000; Boiko et al., 2006). In differentiating myoblasts, the cell cycle inhibitor diacylglycerol kinase zeta (DGK-zeta) arrests cell cycle by inhibiting *cyclin D1* expression and increasing *PC3*/*Tis21*/*BTG2* expression (Evangelisti et al., 2009). On the other hand, in B cells PRMT1 complexed with BTG2 methylates cyclin-dependent kinase 4 (CDK4), inhibiting the formation of a CDK4-Cyclin D3 complex and G1 cell cycle progression (Dolezal et al., 2017).

Of note, *cyclin D1* suppression appears to be the preferential but not the exclusive mechanism for the *Tis21*/*BTG2*-mediated G1 arrest, since this occurs also by reducing *cyclin E* and *CDK4* levels in 293 cells, which are devoid of functional Rb, p53 and cyclin D1 proteins (Lim et al., 1998). However, in this important demonstration of cyclin-D–independent mechanisms results may not fully generalize, as 293 cells are highly transformed.

Interestingly, it appears that BTG2 (and Tob1, but not other BTG/Tob proteins) is able in MCF-7 cells to inhibit proliferation, i.e., the progression to the S-phase, also through interaction with the Caf1a and Caf1b deadenylase enzymes (Doidge et al., 2012), as part of CCR4-NOT complexes (Morel et al., 2003). In these studies revealing an alternative posttranscriptional regulatory mechanism, the functional *in vivo* relevance remains to be fully established.

Furthermore, *PC3*/*Tis21*/*BTG2* has also been implicated in the inhibition of the G2/M phase of the cell cycle, primarily in monocyte cells (Kim et al., 2014), in DNA-damaged mouse embryonic stem cells (Rouault et al., 1996) and in tumor cells. Accordingly, *PC3*/*Tis21*/*BTG2* upregulation correlates with G2/M arrest in U937 monocytic tumor cells by blocking cyclin B1-Cdc2 binding p53-independently (Ryu et al., 2004); in a similar manner, *PC3*/*Tis21*/*BTG2* prevents transformed hepatocytes from proliferating by interfering with cyclin B1-cdk1 activity (Park et al., 2008). Moreover, during senescence *BTG2* expression increases, and *BTG2* is able to arrest cell cycle by inducing replicative senescence p53-independently in human fibroblasts through its ability to bind and sequester the cell cycle regulator peptidyl-prolyl isomerase Pin-1 (Wheaton et al., 2010).

**FIGURE 1:**
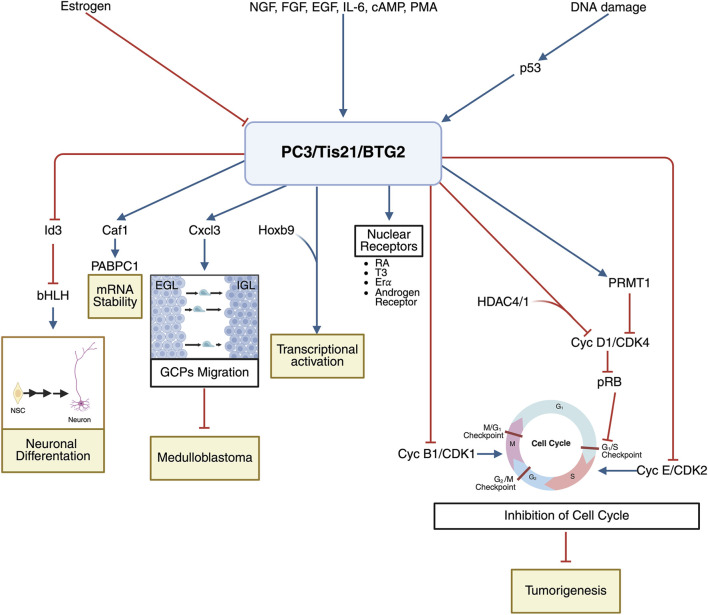
Schematic representation of the *PC3/Tis21/BTG2* mechanism of action. Upper section: Upstream regulators of *PC3/Tis21/BTG2* expression. Growth factors, signaling molecules, and DNA damage (via p53) activate *PC3/Tis21/BTG2* transcription, whereas estrogen represses it. Lower section: Main molecular targets, interactors, and biological functions of *PC3/Tis21/BTG2*. - Neuronal differentiation: Inhibits *Id3*, relieving repression of bHLH factors and promoting neuronal differentiation in neurogenic niches. - mRNA stability: Interacts with Caf1 deadenylase and PABPC1 to promote posttranscriptional mRNA deadenylation. - GCP migration: Upregulates *Cxcl3*, supporting GCP migration from the EGL to the IGL and limiting MB onset. - Transcriptional activation: Modulates transcription through interaction with Hoxb9. - Cell cycle inhibition: Blocks cyclin B1/CDK1 in G2/M; represses *cyclin D1* transcription via HDAC1/4 and reduced pRb activity at G1/S; decreases cyclin E/CDK2 activity. These effects contribute to its tumor-suppressor function. - Nuclear receptor interactions: Binds several nuclear receptors (T3, RAR, ERα, androgen receptor). Abbreviations: EGL, external granular layer; ERα, estrogen receptor α; GCP, granule cell precursor; IGL, internal granular layer; NSC, neural stem cell; NGF, nerve growth factor; RA, all-trans retinoic acid; T3, triiodothyronine. Created in BioRender: https://BioRender.com/j48k0w5.

As a whole, this indicates that *PC3*/*Tis21*/*BTG2* can exert cell cycle arrest through different pathways, depending on the cellular context, and it behaves as a tumor suppressor, being able to inhibit cell cycle even after pRb inactivation, a common feature of tumorigenesis. Nonetheless, many mechanistic insights derive from overexpression or single-line studies, and the relative dominance of each pathway in physiological conditions remains partially unresolved. See Figure 1.

Moreover, the cell cycle length is a flexible parameter modifiable by physiological or applied stimuli, as examples linked to *PC3*/*Tis21*/*BTG2* and *BTG1* show. According to Calegari et al. (2005), telencephalic progenitors undergoing neuron-generating division, labeled by *Tis21*-*GFP* expressing, show a longer cell cycle (Calegari et al., 2005). On the other hand, when a neurogenic stimulus (such as voluntary running) is applied, capable of counteracting the deficit of neurogenesis occurring in *BTG1* knockout mice, dentate gyrus progenitors and stem cells show an increase of proliferation and a shorter cell cycle S-phase (Farioli-Vecchioli et al., 2014b).

Finally, there are multiple examples of antiproliferative activity of *BTG2* in different tumors and different cell types, namely, neuroblastoma (Satoh et al., 2025), medulloblastoma (Farioli-Vecchioli et al., 2007), lung adenocarcinoma (He Y et al., 2024), cardiomyocyte (Velayutham et al., 2023), renal cell carcinoma (Qi et al., 2022), colorectal cancer (Li et al., 2021), ovarian cancer (Wang et al., 2021), esophageal squamous cell carcinoma (Guo et al., 2021), oral squamous cell carcinoma (Lee J et al., 2015), breast cancer cells (Devanand et al., 2019; Zhang et al., 2013), hepatocellular carcinoma (Jiang et al., 2018; Huang et al., 2017; Zhang et al., 2011), pre-B cells (Dolezal et al., 2017), thyroid cancer cells (Zhao and Pang, 2017), skin cancer cells (Gao et al., 2016), human renal carcinoma cells (Sima et al., 2016), monocytes (Kim et al., 2014), prostate carcinoma cells (Chiang et al., 2014; Chung et al., 2012), gastric cancer cells (Zhang et al., 2010), murine B lymphoma cells (Hata et al., 2007; also *BTG1* arrested cell cycle), human leukemia cells U937 (Kwon et al., 2005). All these observations of antiproliferative activity of *BTG2* in different tumors stand for its broad tumor suppressor activity, though many analyze only one cancer cell line at a time and rely on short-term proliferation assays.

Similarly, there are examples of deregulated cell cycle and *BTG2*: in human laryngeal carcinoma (Liu et al., 2009), breast cancer (Möllerström et al., 2010; increased survival in cancers with upregulated *BTG2*), human prostatic carcinoma cells (Tsui et al., 2008).

### 5.2 Apoptosis and *PC3*/*Tis21*/*BTG2*

Concerning apoptosis, it has been shown that *PC3*/*Tis21*/*BTG2* is in general antiapoptotic either in neural cells (Corrente et al., 2002; Micheli et al., 2015; el-Ghissassi et al., 2002) and in tumors, where *BTG2* can prevent apoptosis, such as in breast cancer cells (Zhang et al., 2013) and in the human hepatocellular carcinoma cell line Huh-7 by blocking the damage signal from p-ATM(S1981) *via* activation of PRMT1 (Choi et al., 2012). The latter is a well-elucidated molecular mechanism, albeit shown in a single model. Conversely, several studies report that *BTG2* promotes apoptosis, such as in gastric cancer cells (Zhang et al., 2010) or after myocardial infarction and in breast cancer, where *BTG2* inhibited the antiapoptotic effects of miR-7-5p or miR-27a-3p, respectively, by inactivation of the PI3K/AKT signaling pathway (Hu et al., 2020; Zhu et al., 2020). Similarly, there is clear evidence that *Tis21*/*BTG2* knockout leads to activation of the PI3K/AKT pathway, leading to reduced apoptosis and increased proliferation in the medulloblastoma mouse model *Ptch1* ^+/−^/*Tis21* ^KO^ (Ceccarelli et al., 2021). This effect of *Tis21*/*BTG2* deletion is observed in background *Ptch1* ^+/−^, suggesting that multiple interactions of *Tis21*/*BTG2* with intermediate targets may account for differences in *Tis21*/*BTG2*-dependent regulation of apoptosis. *BTG2* also mediates the proapoptotic effect of C-reactive protein in monocytes (Kim et al., 2014), and *Tis21*/*BTG2* induces apoptosis in U937 human lymphoma cancer cells after EGF-induced phosphorylation and consequent activation of the mitotic regulator Pin-1 (Hong et al., 2005). Finally, *BTG2* favors retinal apoptosis by inducing the proapoptotic factor Bax in a guinea pig myopic model and is counteracted by miR-92b-3p intravitreal injection, which reduces *BTG2* expression, thereby ameliorating also DNA damage (Liu et al., 2024).

Thus, it appears that the dual pro-survival and pro-apoptotic roles of *BTG2* arise from its ability to engage distinct signaling modules with opposing functional outputs. When BTG2 interacts with PRMT1 and modulates the ATM damage-response pathway, it stabilizes cells against apoptosis, as observed in neural cells and hepatocellular carcinoma. Conversely, in contexts where *BTG2* suppresses pro-survival PI3K/AKT signaling–often through antagonism of specific microRNAs–or where it activates apoptosis-effectors such as *Pin-1* or *Bax*, *BTG2* becomes a promoter of cell death. Moreover, its apoptotic role is strongly conditioned by the genetic background, as illustrated by the *Ptch1* ^*+/−*^ medulloblastoma model, where *BTG2* deletion paradoxically enhances cell survival. Therefore, the survival outcome reflects the dominance of the particular *BTG2*-dependent pathway engaged within the specific cellular and signaling milieu.

### 5.3 Negative regulation of cell cycle by *BTG1*

**FIGURE 2:**
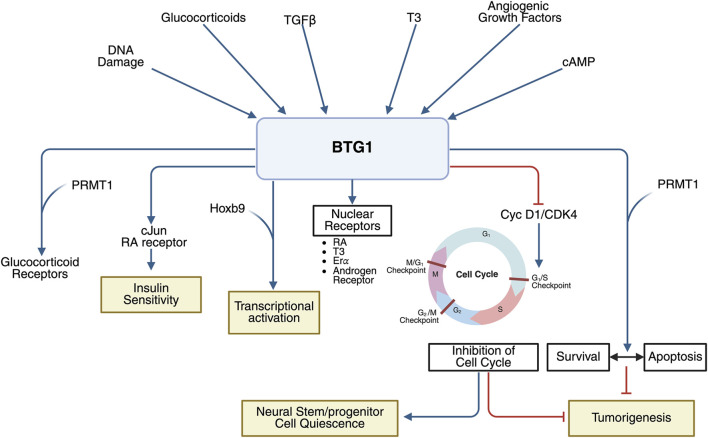
Schematic representation of the *BTG1* mechanism of action. Upper section: molecules and pathways regulating the expression of *BTG1*. *BTG1* is activated by glucocorticoids, TGFβ, T3, angiogenic growth factors, cAMP, and DNA damage. Lower section: *BTG1* molecular targets and interactors, as well as role of *BTG1* in controlling distinct biological outputs. - Insulin sensitivity improvement: by promoting *c-Jun* expression through stimulation of c-Jun and RA receptor activities. - Glucocorticoid receptors: Activates the signaling by interacting with PRMT1. - Transcriptional regulation: Modulates transcription via interaction with Hoxb9. - Nuclear receptor interactions: Binds several nuclear receptors (T3, RAR, ERα, androgen receptor). - Neural stem/progenitor cell quiescence: Maintains the undifferentiated pool of stem/progenitor cells. - Cell cycle inhibition: Inhibits *cyclin D1* transcription and represses cyclin D1/CDK4 activity. - Cell survival and apoptosis: Balances proliferative and death signals, interacting with PRMT1. Control of cell cycle and apoptosis contributes to its tumor-suppressor function. Abbreviations: RA, all-trans retinoic acid receptor; T3, triiodothyronine receptor; Erα, estrogen receptor α. Created in BioRender: https://BioRender.com/ldvmlvk.

The initial evidence that *BTG1* had antiproliferative activity came from experiments of overexpression in NIH3T3 cells (Rouault et al., 1992). Moreover, *BTG1* expression is maximal in the G0/G1 phases of the cell cycle and is downregulated when cells progress to G1 (Rouault et al., 1992), similarly to what occurs for *PC3*/*Tis21*/*BTG2* after NGF or serum stimulation (Montagnoli et al., 1996). Furthermore, we demonstrated that *BTG1* negatively regulates the proliferation of cerebellar granule neuron precursor cells by inhibiting the expression of *cyclin D1* (Ceccarelli et al., 2015). See Figure 2.

Further evidence that overexpression of *BTG1* negatively regulates cell proliferation in several cell types was in macrophages (Suk et al., 2015), erythroid colonies (Bakker et al., 2004), microglia (Lee et al., 2003), quail myoblasts (Marchal et al., 1995; Rodier et al., 1999), endothelial cells (Iwai et al., 2004), ovary cells (Li et al., 2009), brain cells (Farioli-Vecchioli et al., 2012b; Ceccarelli et al., 2015), cardiomyocytes (Velayutham et al., 2023). Yet, most of the several non-neural tissues, use single-model systems and would need *in vivo* validation.

A growth arrest by *BTG1* may be also associated with induction of terminal differentiation, as in myoblasts (Rodier et al., 1999) and erythroid progenitors (Bakker et al., 2004), and with heightened apoptotic frequency, as observed in NIH3T3 cells (Corjay et al., 1998), in brain glioma cells (Qian et al., 2019), and in microglia (Lee et al., 2003), or in esophageal squamous carcinoma cells (Sun et al., 2014c).

Furthermore, the *in vivo* ablation of *BTG1* causes an increase of proliferation of stem and progenitor cells in the early postnatal (day 7) dentate gyrus and subventricular zone (SVZ), while at older stages stem and progenitor cells of both regions show reduced proliferation, which can be rescued by neurogenic stimuli (Farioli-Vecchioli et al., 2012b; Micheli et al., 2018; Mastrorilli et al., 2017). This also indicates that neural stem cells retain plasticity in cell cycle and self-renewal control also in old age. Interestingly, in the adult neurogenesis of hippocampal dentate gyrus, the ablation of *BTG1* causes also massive apoptosis of stem cells, evidently the consequence of an uncontrolled proliferation, as described in Section 6.2 “Role of *BTG1* in adult neurogenesis” (Farioli-Vecchioli et al., 2012b). Altogether, this suggests that a *BTG1*-dependent disinhibition or, conversely, a strong inhibition of cell cycle may lead to apoptosis, due to a conflict between growth signals (Pucci et al., 2000).

### 5.4 Apoptosis and *BTG1*

*BTG1* expression has been shown to be deregulated and/or be responsible for cell cycle arrest and altered apoptosis in different types of cancer: in hepatocellular carcinoma (Li X et al., 2023), medulloblastoma (Ceccarelli et al., 2020a), endometrial carcinoma (Li et al., 2020), glioblastoma (Wang et al., 2019), cervical cancer (Zhu and Han, 2019), colon cancer (Su et al., 2019), colorectal cancer (Su et al., 2019; Zhao et al., 2017), glioma cancer (Qian et al., 2019), bladder cancer (Nan et al., 2016), kidney cancer (Sun et al., 2015), gastric cancer (Zheng et al., 2015), renal cell carcinoma (Liu et al., 2015), thyroid carcinoma (Lu et al., 2014), non-small cell lung cancer (Sun et al., 2014a), hepatocellular carcinoma (Sun et al., 2014b), nasopharyngeal carcinoma (Sun et al., 2014d), esophageal squamous cell carcinoma (Sun et al., 2014c), ovarian cancer (Zhao et al., 2013), breast cancer (where *BTG1* inhibits cell cycle and also metastasis; Zhu et al., 2013; Li W et al., 2014; Sheng et al., 2014). Across cancers, *BTG1* consistently appears as a relevant regulator, though many studies rely on endpoint assays and lack long-term tumorigenesis models.

In summary, *PC3*/*Tis21*/*BTG2* and *BTG1* play a key role as negative regulators of cell cycle mainly through G1 inhibition of *cyclin D1*, but also through G2/M pathways. Both genes have an impact on cell survival.

*BTG2* and *BTG1* display opposite pro- or anti-survival effects because both function as context-dependent modulators of cell cycle restraint and differentiation, whose impact varies with the molecular environment of each cell type or tumor. Their shared core activity — inhibiting G1 progression through cyclin-D-dependent mechanisms — can either stabilize cells by reducing replication stress or trigger apoptosis when this arrest conflicts with strong mitogenic or oncogenic signals.

For *BTG2*, survival outcomes depend on which downstream pathway predominates. The apoptotic balance is further shaped by its integration with differentiation cues, by microRNA networks and by genetic context, as shown by genotype-specific effects in medulloblastoma.

*BTG1* follows similar principles: depending on a tumor’s reliance on continuous cycling, its differentiation state, and sensitivity to cell cycle interruption, *BTG1*-mediated G1 inhibition can have protective or cytotoxic consequences. Thus, the pro- or anti-survival nature of *BTG2* and *BTG1* emerges from how their cell cycle and differentiation functions intersect with the specific genetic and signaling landscape of each cellular context.

## 6 Regulation of neurogenesis and neural differentiation by *PC3*/*Tis21*/*BTG2* and *BTG1*

*BTG1* and *BTG2*, due to their roles in regulation of cell cycle and apoptosis, are crucial for both embryonal and adult neurogenesis — the process by which new neurons are generated from neural stem and progenitor cells.

Focusing on adult neurogenesis, this persists throughout life from stem cells present in two primary niches: the subgranular zone (SGZ) of hippocampal dentate gyrus and the SVZ adjacent to the lateral ventricles (Kempermann et al., 2015; Lim and Alvarez-Buylla, 2016). In both the neurogenic niches, radial glia-like neural stem cells (RGLs) progress through sequential stages of maturation, ultimately differentiating into fully integrated neurons within their respective circuits, contributing to functions such as learning, memory, and olfactory processing. In the hippocampus, this process is critical particularly for pattern separation–the ability to distinguish similar memory representations–supported by the integration of newly generated neurons into existing dentate gyrus circuits (Aimone et al., 2011; Sahay et al., 2011; Farioli-Vecchioli et al., 2008).

Within the dentate gyrus, RGL stem cells reside in the SGZ and express *GFAP*, *Nestin*, and *Sox2* (Seri et al., 2001; Komitova and Eriksson, 2004; Steiner et al., 2006). These neural stem cells generate proliferative neural progenitor cells (expressing *Nestin, Sox2, DCX*), and then neuroblasts (*DCX* ^+^; Filippov et al., 2003; Fukuda et al., 2003; Kronenberg et al., 2003; Steiner et al., 2006). These mature into postmitotic granule neurons co-expressing *DCX* and *NeuN* and ultimately differentiate into fully mature neurons expressing *calbindin* and *NeuN* (Brandt et al., 2003; Steiner et al., 2004). Similarly, SVZ, the other adult neurogenic brain region (Alvarez-Buylla and Lim, 2004), contains type B stem astrocytic-like cells, type C transit amplifying cells and type A migrating neuroblasts (Lagace et al., 2007; Zhao et al., 2008). These developmental stages, identified by distinct molecular markers, have been primarily characterized in murine models.

Many studies have investigated the mechanisms that regulate the balance between quiescence and activation of neural stem cells, raising questions about whether the neural stem cell pool is progressively depleted with aging or can be reactivated by neurogenic stimuli, and if adult neurogenesis occurs also in human.

Two major hypotheses have been proposed to explain neural stem cell self-renewal in the SGZ of the adult dentate gyrus. The recurrent self-renewal model suggests that NSCs alternate between quiescence and activation, undergoing multiple asymmetric divisions, giving rise to neurons or astrocytes, or symmetric divisions before returning to a dormant state; this model preserves the stem cell pool and sustains neurogenesis throughout life (Bonaguidi et al., 2011; Urbán et al., 2016; Obernier et al., 2018; Pilz et al., 2018; Ceccarelli et al., 2020b). Conversely, the disposable stem cell model suggests that once activated, NSCs divide asymmetrically several times and then terminally differentiate into neurons or astrocytes, ultimately depleting the stem cell reservoir (Encinas et al., 2011). Recent evidence indicates that these models may represent age-dependent strategies: rapid proliferation consistent with the disposable model predominates early postnatally, whereas adulthood and aging favor neural stem cell pool maintenance and quiescence (Harris et al., 2021; Ibrayeva et al., 2021; Martín-Suárez and Encinas, 2021). Neural stem cell also exhibit heterogeneity: Nestin-positive cells have longer lifespans and lower division rates, while *Ascl1*-expressing cells are more activation-prone (Ibrayeva et al., 2021). These findings support the existence of distinct neural stem cell subpopulations with dynamic activation and self-renewal behaviors shifting over time.

Other single-cell RNA-seq studies indicate continuous low-frequency generation from stem/progenitor cells of new neurons with prolonged maturation, leading to accumulation of immature granule neurons (Zhou et al., 2022; review Micheli et al., 2025). Recently, as the latest step of a debate about whether adult neurogenesis occurs in humans, RNA-seq data indicated that adult neurogenesis also occurs in the human dentate gyrus from stem/progenitor cells (Dumitru et al., 2025). This has profound implications for translational research, since strategies to modulate neurogenesis could become treatments for neurodegenerative diseases and age-related cognitive decline.

### 6.1 Role of *PC3*/*Tis21*/*BTG2* in embryonic and adult neurogenesis

Numerous *in vivo* investigations have demonstrated that *BTG2* is expressed in neural progenitor cells undergoing a neurogenic asymmetric (neuron generating) division (Iacopetti et al., 1994; 1999; Malatesta et al., 2000; Haubensack et al., 2004; Calegari et al., 2005; Attardo et al., 2010). Using a *Tis21*-*GFP* reporter mouse model, early-born neurons were shown to express *Tis21*-*GFP*, thereby marking sites of endogenous *Tis21* expression in the neural tube, ventricular zone and cortex (Kowalczyk et al., 2009; Attardo et al., 2010). A strength of these early studies is their consistent use of embryonic tissue and lineage markers; however, several rely on static imaging of fixed tissue, limiting temporal resolution of asymmetric divisions. See Table 1 and Figure 1. Remarkably, as revealed by direct overexpression *in vivo*, *BTG2* causes brain progenitor cells to differentiate (Canzoniere et al., 2004; Farioli-Vecchioli et al., 2008). *In vivo*, as shown by a transgenic mouse expressing *PC3*/*Tis21*/*BTG2* driven by the neural progenitor marker *Nestin* (Tg *Nestin*-rtTA-TRE-*PC3*), *BTG2* induces the full differentiation of neural progenitor cells, including neuroblasts in the neural tube at P12 and the cerebellum granule precursors during early postnatal development, as well as in adult progenitor cells of the dentate gyrus and of the SVZ (Canzoniere et al., 2004; Farioli-Vecchioli et al., 2008). *In vitro*, *BTG2* synergizes with NGF to promote differentiation in the neuronal PC12 cell line (Corrente et al., 2002; el-Ghissassi et al., 2002). See Table 2.

**TABLE 1:** Localization of *PC3*/*Tis21* expression in early and postnatal neurogenesis.

Brain ventricular zone at E10-E14 and adult neurogenic niches				
Model used	Developmental stage/Region and cell type observed	Morphology/phenotype	Cellular correlations	Reference
Wild-type rat	Neuroepithelial (NE) cells of the ventricular zone of brain embryo (E10-14)	*PC3* is expressed in embryonic brain vesicles along a caudo-rostral gradient, corresponding spatially and temporally to the gradient of neurogenesis	- *PC3* expression in all CNS regions is restricted to the ventricular zone of the NE - neuronal precursors cease to express *PC3* when they migrate to the mantle zone	Iacopetti et al. (1994)
Wild-type mouse	NE cells of the ventricular zone of brain embryo (E10-E14)	*Tis21* mRNA is transiently expressed in the NE cells during the G1 phase of the cell cycle	*Tis21* is expressed in NE cells that will generate postmitotic neurons at their next division, but not in proliferating NE cells	Iacopetti et al. (1999)
Dissociated telencephalic vesicles from E15 wild-type rat embryos	Primary culture cells of telencephalic vesicles and NIH3T3 cell line	BrdU expression becomes diluted during subsequent divisions	- Cortical precursor clones transduced with *PC3* show an asymmetric pattern of BrdU dilution - *PC3* overexpression reduces the proliferation rate in both NIH3T3 cells and cortical precursor cells	Malatesta et al. (2000)
*Tis21*-*GFP* expressing (constitutive) knockin mouse	NE cells of the E9-E12 brain embryo	*Tis21* is expressed in forebrain (telencephalon), midbrain and hindbrain neuroepithelium	*Tis21* marks apical, asymmetric divisions of NE cells generating another NE cell and a neuron, and also symmetric divisions of basal progenitors generating two neurons	Haubensak et al. (2004)
*Tis21*-*GFP* expressing (constitutive) knockin mouse	Telencephalic progenitor cells (E10.5 and E14.5)	*Tis21* is expressed in telencephalic progenitors undergoing neuron-generating division	Telencephalic progenitors undergoing neuron-generating division, identified by labeling with *Tis21*-*GFP* cumulative BrdU labeling, show a longer cell cycle	Calegari et al. (2005)
*Tis21*-*GFP* expressing (constitutive) knockin mouse	Intermediate neural progenitors of cerebral cortex	*Tis21* is expressed in intermediate neural progenitors (INPs) of cerebral cortex	Neurogenic divisions of INPs of cerebral cortex were labeled by *Tis21*-*GFP* mature into neurons for preplate, deep, and superficial layers	Kowalczyc et al. (2009)
*Tis21*-*GFP* expressing (constitutive) knockin mouse	Adult hippocampus stem/progenitor cells and neurons	*Tis21* is expressed in progenitor cells and in immature and mature neurons	*Tis21* expression, detected by GFP, is bimodal - in progenitor cells - in immature new neurons and in terminally differentiated neurons → Suggestion for a role of *Tis21* in terminal differentiation	Attardo et al. (2010)

BrdU: bromodeoxyuridine; E: embryonic day; GFP: green fluorescent protein; INP: intermediate neural progenitor; NE: neuroepithelium.

**TABLE 2:** Effect of *PC3*/*Tis21*/*BTG2* overexpression or knockout on neural differentiation and neurogenesis.

Overexpression or silencing	Cell type genetically modified	Modified morphology/phenotype	Cellular and molecular changes	References
*PC3/Tis21/BTG2 - In vitro *models: NIH3T3 and PC12 cells				
*PC3*/*Tis21* overexpression	NIH3T3 cells and PC12 cells transduced with retrovirus (pBABE Neo vector)	Increased production of new neurons in the neural tube at E12.5	In NIH3T3 and in PC12 cells the overexpression of *PC3*/*Tis21*/*BTG2* - impairs the transition from G1 to S phase, reducing proliferation, as detected by BrdU incorporation time-course, colony formation assay, and flow-cytometry analysis of cell cycle phases - impairs the pRb phosphorylation, indicating cell cycle arrest	Montagnoli et al. (1996)
*PC3*/*Tis21* overexpression	NIH3T3, *Rb* ^−/-^ NIH3T3 cells, and *cyclin D1* ^−/−^ mouse embryo fibroblasts	Induction of pRb dephosphorylation by *PC3* and reversal by cyclins	*PC3*/*BTG2* impairs the G1-S transition by inhibiting pRb function in consequence of a downregulation of *cyclin D1* levels: this action requires the GR/A box	Guardavaccaro et al. (2000)
*PC3*/*Tis21* overexpression or silencing	PC12 cells (plasmid-transfected)	Increased expression of tyrosine hydroxylase and Neurofilament 160 kDa	*PC3*/*BTG2* synergizes with NGF - to promote differentiation in the neuronal PC12 cell line (significant increase of tyrosine hydroxylase and Neurofilament 160 kDa) - to protect against apoptosis induced by NGF deprivation Antisense *PC3*/*BTG2* triggers apoptosis	Corrente et al. (2002)
*BTG2* overexpression or silencing	*BTG2*-inducible PC12 cell clones	Increased number of neurites	- *BTG2* overexpression in PC12 cells enhanced the NGF-induced differentiation - Apoptosis of NGF-differentiated cells was triggered by a *BTG2* antisense nucleotide	el-Ghissassi et al. (2002)
*PC3*/*Tis21* - *In vivo* models				

Overexpression or knockout	Cell type genetically modified	Modified morphology/phenotype	Cellular and molecular changes	References
*PC3/Tis21 - Neural tube at E12.5 (cervical level)*				
*PC3*/*Tis21* overexpression	Transgenic mouse: nestin-driven *PC3*/*Tis21* expression in neural tube cells since conception	Increase of new neurons produced in the neural tube at E12.5	*PC3*/*Tis21* transgenic mouse show - in the neural tube, enhanced neural differentiation: increased number of new neurons (*Tis21* ^+^/*βIII tubulin* ^+^ and *MAP-2* ^+^ cells) - in the VZ of the neural tube, inhibition of new neurons cell cycle (reduced BrdU incorporation)	Canzoniere et al. (2004)
*PC3/Tis21 - Adult dentate gyrus (DG) stem and progenitor cells (P60)*				
*PC3*/*Tis21* overexpression	Transgenic mouse: nestin-driven *Tis21* expression in stem/progenitor cells (transgene activated in the period P30-P95)	- Accelerated differentiation of new neurons (with reduced dendritic length and spine density) - No significant morphological change in hippocampus or DG volume	*Tis21*-overexpressing mice show - accelerated differentiation of immature and mature new neurons (1-5- and 28-day-old) - inhibition of cell cycle in stem and progenitor cells without difference in total BrdU incorporation - reduced LTP of DG neurons - reduced spatial and associative memory → The timing of differentiation influences the function of new neurons	Farioli-Vecchioli et al. (2008)
Tis21 knockout (constitutive)	Stem/progenitor cells/neurons	- No significant morphological change in hippocampus or DG volume - Specific impairment of the terminal differentiation of DG neurons	*Tis21*-null mice show impaired terminal differentiation - increased number of immature neurons and decrease of mature neurons - increased expression of the inhibitor of neural genes *Id3*, due to the lack of *Tis21* inhibitory binding to the promoter of *Id3* - acceleration of cell cycle (increased number of DG progenitor cells entering in S-phase in *Tis21*-null mice; reduced length of G1 phase progenitor cells) - reduced associative memory	Farioli-Vecchioli et al. (2009)
Tis21 knockout (constitutive)	Stem/progenitor cells/neurons	- Neurogenic stimuli (running or fluoxetine) increase the generation of new neurons in *Tis21* ^KO^ DG but do not restore the reduced differentiation rate - Overexpression of *NeuroD2* or silencing of *Id3* rescues impaired differentiation in the DG	- Defective differentiation rate of *Tis21* ^KO^ neurons is not rescued by neurogenic stimuli (running or fluoxetine), despite their ability to increase the generation of new neurons in DG - Overexpression of *NeuroD2* or silencing *Id3* rescues the defective rate of differentiation → *Id3* and *NeuroD2* control the terminal differentiation pathway with *Tis21*, and they are target of *Tis21*; *Id3* is also transcriptionally regulated by *Tis21* (Farioli-Vecchioli et al., 2009) - Treatment with fluoxetine significantly reduces the length of the S phase in *Tis21* knockout 2-month-old DG progenitor cells	Micheli et al. (2017a)
*PC3/Tis21 - Adult subventricular zone stem cells and neurons (P60)*				
Tis21 knockout (constitutive)	B Stem cells/C transient amplifying cells/A neuroblasts	Reduced number of differentiated SVZ-derived neurons in olfactory bulb	- *Tis21*-null SVZ A neuroblasts show impaired terminal differentiation - *BMP4* or *Id3* silencing restores the differentiation defect, suggesting that increased *Id3* levels and decreased *BMP4* levels are at the origin of this defect - Reduced number of terminally differentiated neurons in SVZ results in a decrease of the neurons migrated in the olfactory bulb and is accompanied by increased olfactory threshold - *Tis21* maintains stem cells in quiescence, since *Tis21*-null proliferating B stem cells increase in number	Farioli-Vecchioli et al. (2014a)
*PC3/Tis21 - Cerebellum: granule cell precursors (GCPs)*				
*PC3*/*Tis21* overexpression (nestin-driven)	Expressed in GCPs since conception until P5 (nestin-driven)	Decrease of cerebellar size (in mice with β-actin-driven *PC3* transgene)	- Increased differentiation (induction of *Neurofilament 160 kDa* and *Math1* expression) - Inhibition of cell cycle (reduced BrdU incorporation, decreased expression of *cyclin D1*)	Canzoniere et al. (2004)
*PC3*/*Tis21* overexpression (β-actin-driven)	Expressed in GCPs since conception until adulthood (β-actin-driven)	Large decrease of cerebellar size (55% decrease in 6%–8% of mice) more pronounced than in the whole brain	- Increased differentiation (induction of *Neurofilament 160 kDa* and *Math1* expression) - Inhibition of cell cycle (reduced BrdU incorporation, decreased expression of *cyclin D1*)	Canzoniere et al. (2004)
*PC3*/*Tis21* overexpression (β-actin-driven)	Expressed in GCPs since the perinatal stage (β-actin driven)	N.D.	- Increased differentiation (*NeuroD1* ^+^ neurons) - Inhibition of cell cycle (reduced BrdU incorporation, decreased expression of *cyclin D1*; no change in *cyclin D2* or *cyclin B1* expression)	Farioli-Vecchioli et al. (2007)
*Tis21* knockout (constitutive)	GCPs, from embryogenesis	N.D.	- Small but significant reduction of differentiation of cerebellar precursors - No effect on cell cycle (unchanged BrdU incorporation in cerebellar precursors) - Strong decrease of migration of cerebellar precursors out of the surface of cerebellum to the internal layers (accompanied by inhibition of *Cxcl3* expression)	Farioli-Vecchioli et al. (2012a)
*PC3/Tis21 - Neocortex*				
*PC3*/*Tis21* overexpression	Expressed in neural stem cells since conception (β-actin-driven)	Decrease of cortex size (30% decrease)	About 6% of mice with *PC3* transgene active since conception presented small size and brain	Canzoniere et al. (2004)
*Tis21* overexpression	Expressed by electroporation in dorsolateral telencephalon of E13.5 embryo	Selective decrease of cortex size	- No evident change of total BrdU incorporation in neocortex progenitor cells - Decrease of neurogenesis specific for cortex neurons; increase of symmetric neurogenic divisions	Fei et al. (2014)
*PC3/Tis21 - Cochlea*				
*Tis21*-*GFP* expressing (constitutive)	Expressed in spiral ganglion cells of the cochlea since E13.5	The development of Rosenthal’s canals was inhibited in *Tis21*-*GFP* expressing mice	*Tis21*-*GFP* expressing mice presented a decrease in spiral ganglion cell counts. One possible explanation is a defective cochlear ganglion neurons’ transition from proliferation to differentiation and maturity	Yamada et al. (2015)

BrdU: bromodeoxyuridine; DG: dentate gyrus; E: embryonic day; GCP: granule cell precursor; GFP: green fluorescent protein; N.D.: not determined; P: postnatal day; VZ: ventricular zone.

Further findings indicate that *BTG2* is necessary for the differentiation of new neurons, as demonstrated in a *BTG2* knockout mouse model where there is impaired terminal differentiation of new neurons in the dentate gyrus and in the SVZ (Farioli-Vecchioli et al., 2009; 2014a). Thus, *BTG2* is a pan-neural gene essential for the development of new neurons produced during adulthood in the main neurogenic regions of the adult brain, the hippocampus and the SVZ (Farioli-Vecchioli et al., 2009; 2014a). *BTG2* expression is also required for the differentiation of spiral ganglion cells in the cochlea (Yamada et al., 2015).

It has been demonstrated that spatial and contextual memory are significantly impaired when the overexpression or deletion of *BTG2* accelerates or delays, respectively, the development of new neurons in the hippocampus, which is crucial for learning and memory (Farioli-Vecchioli et al., 2008; 2009). This implies that *BTG2* controls the timing of the new neuron’s recruitment into memory circuits and that the amount of time the newborn neurons spend in various stages of neuronal differentiation is crucial for their eventual participation in learning and memory (Farioli-Vecchioli et al., 2008; 2009). Similarly, deletion of *PC3*/*Tis21*/*BTG2* impairs differentiation of olfactory bulb neurons of the SVZ, in association with a loss of olfactory discrimination (Farioli-Vecchioli et al., 2014a). All this suggests as a general paradigm that the timing of differentiation of a neuron is critical for its function.

This need for *BTG2* in neuron maturation is in line with the fact that *BTG2* is expressed in the proliferating neuroblasts of the neural tube ventricular zone and, to a lesser degree, in the mantle zone differentiating neuroblasts, during brain development. Postnatally, *BTG2* is expressed in cerebellar precursors primarily in the external granular layer (i.e., the proliferating region of the neuroepithelium) and in the hippocampus in proliferating and differentiating progenitor cells (Iacopetti et al., 1994; Canzoniere et al., 2004; Farioli-Vecchioli et al., 2008; Attardo et al., 2010). *BTG2*’s prodifferentiative effect seems to result from both a BTG2-dependent activation of proneural genes in brain progenitor cells and a suppression of cell cycle progression by inhibition of *cyclin D1* expression (Canzoniere et al., 2004; Farioli-Vecchioli et al., 2009). Remarkably, all *in vivo* models of *PC3/Tis21/BTG2* show a complete convergence of findings. This is true both for the reporter model (*Tis21-GFP*), which reveals the localization of *Tis21/BTG2*, and for the transgenic overexpression models in neural stem/progenitor cells and the constitutive knockout model, which respectively accelerate or delay neural differentiation. Indeed, BTG2 activates proneural genes by binding to the promoter of *Id3*, a crucial inhibitor of proneural gene activity, and by adversely controlling its function (Farioli-Vecchioli et al., 2009). In fact, *BTG2* acts as a transcriptional cofactor, as it interacts with and controls the promoters not only of *Id3* but also of *cyclin D1*, *RAR-β* and *PRMT1* as part of transcriptional complexes (Farioli-Vecchioli et al., 2007; Passeri et al., 2006; Lin et al., 1996). The defective differentiation of *Tis21*-null SVZ neurospheres is rescued by the silencing of the anti-differentiative gene *Id3*, or by the treatment with the prodifferentiative molecule BMP4, suggesting that *PC3*/*Tis21*/*BTG2* is an upstream controller of *Id3* and *BMP4* (Farioli-Vecchioli et al., 2014a). Similarly, overexpression of *NeuroD2* or the silencing of *Id3* in the dentate gyrus of *Tis21* knockout mice rescues the defective differentiation of hippocampal neurons (Micheli et al., 2017a).

Additionally, neurogenic stimuli such as running or the antidepressant fluoxetine increase the generation of new neurons in *Tis21* ^KO^ dentate gyrus but do not change the reduced differentiation rate (Micheli et al., 2017a).

In summary, *BTG2* primarily regulates the terminal differentiation of brain progenitor cells in the dentate gyrus and SVZ during adult neurogenesis (see Micheli et al., 2015, for a review). In cerebellum, *BTG2* is chiefly needed to regulate the migration and differentiation of the precursor cells of cerebellar granule neurons during the early postnatal development of the cerebellum (Farioli-Vecchioli et al., 2012a).

### 6.2 Role of *BTG1* in adult neurogenesis

Conversely, the chief action of *BTG1* in neurogenesis is to maintain the stem cell pool in quiescence and to prevent its depletion by inhibiting the proliferation of adult stem cells in the dentate gyrus and SVZ, as shown by *in vivo* knockout models as well as by neurosphere cultures (Farioli-Vecchioli et al., 2012b; Tirone et al., 2013). See Table 3 and Figure 2. Interestingly, the deletion of *BTG1 in vivo* causes initially, at an early postnatal stage, hyperproliferation of stem/progenitor cells, followed by slow proliferation and apoptosis at later stages (Farioli-Vecchioli et al., 2012b). It turns out that a neurogenic stimulus such as running fully rescues this defective neurogenesis in both the dentate gyrus and the SVZ, by shortening the S-phase length and the total cell cycle duration of both neural stem (GFAP/Sox2-positive) and progenitor (NeuroD1-positive) cells (Farioli-Vecchioli et al., 2014b; Mastrorilli et al., 2017). Overall, these findings suggest that stem and progenitor cell self-renewal is a plastic process, capable of being reactivated even after periods of quiescence.

**TABLE 3:** Effect of BTG1 knockout on neurogenesis.

BTG1 - In vivo models				
Knockout	Cell type genetically modified	Modified morphology/phenotype	Cellular and molecular changes	Reference
*BTG1 - Adult DG stem and progenitor cells (P60)*				
*BTG1* knockout (constitutive)	Stem/progenitor cells/neurons	Deletion of *BTG1* causes hyperproliferation of DG cells at an early developmental stage (P7), followed by reduced proliferation and apoptosis at a later stage (P60); this occurs also in SVZ	Ablation of *BTG1*, after an hyperproliferative burst at early post-natal stages, reduces the pool of dividing adult stem and progenitor cells in the DG and SVZ by decreasing their proliferative capacity → *BTG1* is required for maintaining adult stem and progenitor cells quiescence and self-renewal	Farioli-Vecchioli et al. (2012b)
*BTG1* knockout (constitutive)	Stem/progenitor cells/neurons	Neurogenic stimulus of running rescues the reduced proliferation of *BTG1*-null DG stem/progenitor cells	- Physical exercise fully rescues the defective reduced proliferation of adult DG stem and progenitor cells, by shortening the S-phase length and the overall cell cycle duration of both neural stem (GFAP-positive and Sox2-positive) and progenitor (NeuroD1-positive) cells - This neurogenic stimulus reactivates the hyperproliferation observed in *BTG1*-null early-postnatal mice and expands the pool of adult neural stem/progenitor cells → The plasticity of neural stem cells devoid of cell cycle inhibitory control can be reactivated by a neurogenic stimulus	Farioli-Vecchioli et al. (2014b)
*BTG1* knockout (constitutive)	Stem/progenitor cells/neurons	Adult DG stem cells with reduced proliferation in the *BTG1* knockout mice are restarted by fluoxetine, or by overexpression in the DG of *Sox2*	- The reduced self-renewal and proliferative capability of adult DG stem cells in the *BTG1* knockout mice are reactivated by fluoxetine, which forces quiescent stem cells to enter the cycle; fluoxetine reactivates proliferation-defective stem cells also in aged *BTG1* knockout mice - Overexpression of *Sox2* in *BTG1* knockout DG cells significantly increases the number of neuroblasts, indicating that *Sox2* is able to promote the self-renewal of proliferation-defective stem cells	Micheli et al. (2018)
*BTG1 - Adult subventricular zone stem cells and neurons (P60)*				
*BTG1* knockout (constitutive)	B Stem cells/C transient amplifying cells/A neuroblasts	Proliferation of adult *BTG1*-null SVZ stem and neuroblast cells is reduced	In adult *BTG1*-null SVZ stem and neuroblast cells the proliferation is reduced with a longer cell cycle and a more frequent entry into quiescence Running restores in *BTG1*-null the normal values of proliferation and cell cycle length and quiescence, without affecting wild-type cells → This suggests that SVZ stem cells are endowed with an additional supply of self-renewal capacity, coupled to cell cycle acceleration	Mastrorilli et al. (2017)
*BTG1 - Cerebellum: granule cell precursors (GCPs)*				
*BTG1* knockout (constitutive)	Postnatal cerebellar granule cell precursors (GCPs)	Proliferation of adult *BTG1*-null precursor cells increases and the adult cerebellar size is greater	*BTG1*-null cerebellar precursor cells continue to proliferate also after P14, when the proliferative zone of the neuroepithelium (EGL) is normally reduced to few layers → *BTG1* is required to restrict the proliferation of cerebellar precursor cells by selectively impairing *cyclin D1* expression	Ceccarelli et al. (2015)

DG: dentate gyrus; EGL: external granular layer; GCP: granule cell precursor; P: postnatal day; SVZ: subventricular zone.

Concerning the molecular mechanism of *BTG1*, a transcriptomic analysis of the dentate gyrus of *BTG1* knockout mice identified 42 genes downregulated by *BTG1* knockout and counter-regulated by physical exercise; among them the most differentially regulated were *alpha-synuclein* (*Snca*), *Fos*, *Arc* and *Npas4*, genes controlling neural proliferation, apoptosis, plasticity and memory (Micheli et al., 2021). In particular, the authors showed that if in *BTG1* knockout mice the low expression of *Snca* is restored, then the defect of neurogenesis is rescued, establishing Snca as a target of *BTG1* that regulates neurogenesis.

Notably, *BTG1* is also required to restrict the proliferation of cerebellar precursor cells by selectively impairing *cyclin D1* expression, as the silencing of *BTG1* does not affect the levels of the other cyclins; moreover, without *BTG1* the adult cerebellum is greater, and less able to coordinate motor activity (Ceccarelli et al., 2015). In absence of *BTG1*, cerebellar precursor cells remain hyperplastic also after postnatal day 14, when the proliferative zone of the neuroepithelium (external granular layer) is normally reduced to a few layers of precursor cells; this opens a window for cell transformation and tumor (Ceccarelli et al., 2015).

As a whole, the main neural action of *PC3*/*Tis21*/*BTG2 in vivo* and *in vitro* is to promote and be required for the differentiation of new neurons, also in adult brain (dentate gyrus and SVZ) and in cerebellum. This action is mediated by regulating *Id3* and also *cyclin D1*, *RAR-β* and *PRMT1* as part of transcriptional complexes. In the cerebellum, *PC3*/*Tis21*/*BTG2* promotes the migration and differentiation of cerebellar precursor cells by inducing the chemokine Cxcl3 (see below). As for *BTG1*, its main neural action is to maintain the stem/progenitor cell pool in quiescence, in the adult brain and in the cerebellum, possibly by regulating *cyclin D1* as well as cohorts of neural genes, including *Snca*.

## 7 Brain tumors involving *PC3*/*Tis21*/*BTG2* and *BTG1*

Despite remarkable advances in cancer research (Bernards et al., 2020; Thakkar et al., 2021), central nervous system (CNS) tumors remain among the most challenging human cancers in terms of resistance to conventional and novel therapeutic approaches, tissue access, morbidity and mortality, with survival rates that have not improved significantly over the past 30 years (Levin et al., 2015; Quader et al., 2022). Brain tumors are usually classified as primary infiltrative (arising from CNS-intrinsic cells) or secondary metastatic tumors, the latter being ten-fold more common and originating from extracranial cancers (mainly lung, breast, skin, or kidney) that spread hematogenously to the brain (Sacks and Rahman, 2020). Metastatic tumors are always malignant, whereas primary brain tumors can be benign or malignant and are classified from 1 to 4 based on the extent of malignancy, according to WHO guidelines (Louis et al., 2021). In adults, primary malignant brain tumors account for only 1%–2% of all tumors, with diffuse gliomas — particularly glioblastoma — being the most common and higly aggessive (Quader et al., 2022; Pouyan et al., 2025). In children and adolescents, CNS tumors are second only to leukemias, with cerebellar medulloblastoma being the most prevalent malignant solid tumor (Ostrom et al., 2018; Huang et al., 2024). Current treatments for brain tumors, based on surgery, radiotherapy, and chemotherapy, have intrinsic limitations and often produce unsatisfactory results; therefore, it is essential to elucidate the mechanisms of tumor progression and therapeutic resistance. In this context, studying the actions of *PC3/Tis21/BTG2* and *BTG1* on the cell biology of brain tumors could be promising. As previously mentioned, *PC3*/*Tis21*/*BTG2* and *BTG1* are expressed in the CNS during development and adulthood, where they regulate key cellular processes, such as cell cycle progression, apoptosis, migration and differentiation. Furthermore, both genes possess tumor suppressor properties. Consistent with this and its close association with other known tumor suppressor genes, such as *Rb* and *p53*, *PC3/Tis21/BTG2* has been extensively studied in malignant brain tumors. By contrast, *BTG1* has been less investigated, despite being recognized, like *PC3/Tis21/BTG2*, as a potential biomarker for the prognosis of cancer patients with various types of solid and hematological tumors.

### 7.1 Glioma

Gliomas are the most common primary tumors of the CNS, accounting for 81% of all malignancies (Ostrom et al., 2014; 2018; Xu et al., 2020). Although they are usually sporadic, a minority of cases are linked to hereditary cancer syndromes, such as neurofibromatosis type 1, Li-Fraumeni syndrome, and Lynch syndrome (Goodenberger and Jenkins, 2012; Ostrom et al., 2014). Gliomas arise predominantly in supratentorial regions, including the frontal, temporal, parietal, and occipital lobes, with few cases occurring elsewhere in the CNS (Larjavaara et al., 2007; Ostrom et al., 2018; Li G et al., 2023). Despite their aggressive and infiltrative nature, extracranial metastases are extremely rare (0.5% of cases), typically involving the spine, vertebrae, lungs, liver, spleen, peritoneum, skin, and lymph nodes (Sun et al., 2017; Kannan et al., 2022). Common clinical symptoms of gliomas include seizures, headaches, and neurological deficits (IJzerman-Korevaar et al., 2018).

Glioma represents a heterogeneous group of highly aggressive tumors derived from glial cells, the non-neuronal cells responsible for neuronal support and protection. Glioma classification is based on glial lineage and includes astrocytomas, oligodendrogliomas, ependymomas, oligoastrocytomas (or mixed gliomas), and some rare or not otherwise specified (NOS) histologies (Ostrom et al., 2018; Lapointe et al., 2018). Recent molecular advances have identified key genetic alterations, such as *IDH* mutations, 1p/19q-codeletion, and H3 Lys27Met, that have been integrated with histopathology to refine glioma classification (Chen et al., 2017; Lapointe et al., 2018; Louis et al., 2021). Adult-type gliomas differ from pediatric gliomas in molecular pathogenesis, prognosis, and therapeutic approaches. Current WHO guidelines classify adult-type diffuse gliomas as: astrocytoma *IDH*-mutant, oligodendroglioma *IDH*-mutant and 1p/19q-codeleted, and glioblastoma (GBM) *IDH*-wildtype (Louis et al., 2021).

GBM accounts for approximately 15% of all primary CNS tumors and 49% of all primary malignant brain tumors; its incidence increases after age 40, peaking between 75 and 84 years (Ostrom et al., 2021; Schaff and Mellinghoff, 2023; Pouyan et al., 2025). It is characterized by rapid cell proliferation, resistance to apoptosis, tendency to necrosis, high invasiveness, angiogenesis, and genomic instability (Furnari et al., 2007). Clinically, GBM can be classified as primary or secondary. The vast majority (∼90%) are primary GBMs, arising *de novo* as grade 4 neoplasms in elderly patients (average age 55), and are genetically characterized by loss of 10q heterozygosity, *EGFR* overexpression or mutation, *MDM2* overexpression, *p16INK4a* deletion, and mutations in the *TERT* promoter and *PTEN* gene. Secondary GBMs, which arise from a pre-existing low-grade diffuse astrocytoma (grade 2) or anaplastic astrocytoma (grade 3), represent approximately 10% of cases, occur in younger adults (≤45 years), and have genetic alterations such as *PDGF*/*PDGFRA* overexpression, loss of 19q heterozygosity, and mutations in the *p53*, *Rb*, and *ATRX* genes (DeAngelis, 2001; Ohgaki and Kleihues, 2007; Hanif et al., 2017; Quader et al., 2022; Pouyan et al., 2025). Thus, several genes and signaling pathways are involved in gliomagenesis, influencing prognosis and therapeutic responses (Cancer Genome Atlas Research Network, 2008; Brennan et al., 2013; Ostrom et al., 2014; Grochans et al., 2022; Pouyan et al., 2025). Among them, methylation of the O^6^-methylguanine-DNA methyltransferase (*MGMT*) promoter is a key prognostic biomarker in GBM. Indeed, methylation of the *MGMT* promoter silences this DNA repair enzyme, preventing removal of alkyl adducts induced by alkylating chemotherapy from the O^6^ position of guanines and hindering its cytotoxic effects (Hegi et al., 2005; Chen et al., 2017; Grochans et al., 2022).

Standard GBM treatment consists of maximal safe surgical resection, followed by radiotherapy and concomitant or adjuvant chemotherapy with temozolomide (TMZ) (Stupp et al., 2005; Karachi et al., 2018; Tan et al., 2020). Despite this optimized treatment regimen, median overall survival remains 15 months (Omuro and DeAngelis, 2013; Hanif et al., 2017; Grochans et al., 2022; Pouyan et al., 2025), although it may reach approximately 23 months in patients with methylated *MGMT* promoter (Berger et al., 2022). Additional chemotherapeutic strategies tested include alkylating agents like carmustine and lomustine, anti-angiogenic agents like anti-VEGF monoclonal antibodies (Bevacizumab), carboplatin, vincristine, anti-FGF antibodies, tyrosine kinase inhibitors, and monoclonal antibodies targeting EGFR (Erlotinib and Gefitinib) (Hanif et al., 2017; Marenco-Hillembrand et al., 2020; Quader et al., 2022). Several immunotherapies are also under clinical investigation worldwide (Yan et al., 2020; Mahmoud et al., 2022; Yasinjan et al., 2023). Despite these treatment options, GBM remains a fatal disease with an extremely poor prognosis. For this reason, experimental research continues to investigate potential molecular targets involved in GBM development and treatment response, with the goal of developing new therapeutic strategies that improve patient survival and quality of life.

In this regard, particular attention has been paid to cell cycle regulatory genes, responsible for cell proliferation and senescence: among these, *Rb* and *p53* are frequently mutated in GBM (Furnari et al., 2007; Yin et al., 2009). As mentioned above, *BTG2* is a p53-inducible gene activated by DNA damage from genotoxic agents (Rouault et al., 1996). Under hypoxia, a pathophysiological feature of low/insufficient oxygen common in solid tumors, p53 also induces *BTG2* expression, promoting apoptosis (Leszczynska et al., 2015). In the tumor context, *BTG2* can act as a proapoptotic gene, since phosphorylation of its Ser^147^ residue by p-Erk1/2 induces the binding to Pin-1, a protein that interacts with the NIMA (Never in mitosis gene A) serine/threonine kinases, resulting in mitochondrial depolarization and increased cell death (Hong et al., 2005). *BTG2* also strongly inhibits cancer cell migration and invasion. In addition to its nuclear and cytoplasmic localization (Varnum et al., 1994), BTG2 localizes in mitochondria, in their inner membrane or matrix, where it negatively regulates ROS generation. In this way, BTG2 inhibits the formation of disulfide bonds in Src, maintaining its cystines in a reduced state and inhibiting its kinase activity, independently of the phosphorylation of its residues Tyr^527^ and Tyr^416^. As a consequence, activation of FAK (focal adhesion kinase), which regulates migration and invasion in many tumor cells through remodeling of actin structure and matrix metalloproteinase-mediated degradation, induced by Src kinase *via* phosphorylation of the Tyr^576^ residue, is inhibited (Lim et al., 2012). All these findings strongly suggest that *BTG2* is a tumor suppressor and plays a role in cell apoptosis, migration, and invasion during cancer development. It is therefore not surprising that *BTG2* is also involved in gliomagenesis. See Table 4 and Figure 1.

**TABLE 4:** *BTG2* and *BTG1* in malignant brain tumors.

Tumor	Model system	Genes/Pathways involved	Results	Reference
*BTG2* in malignant brain tumors				
Glioma (oligodendroglioma)	PDGF-B-induced glioma mouse model	*PDGF-B*	Downregulation of *BTG2* is involved in the acquisition of tumorigenic potential by PDGF-B-induced gliomas, i.e., in the progression of low- to high-grade gliomas	Calzolari et al. (2008)
Glioma (oligodendroglioma)	PDGF-B-induced glioma mouse model	*PDGF-B*; *Cyclin D1*	In PDGF-B-induced glioma cells, overexpression of *BTG2* causes a ten-fold reduction in *cyclin D1* mRNA levels, without modifying the fraction of proliferating or apoptotic cells	Appolloni et al. (2012)
Glioma (GBM)	• U87 and U251 cells • GBM mouse model with U87 cells	miR-27a	miR-27a negatively regulates endogenous *BTG2* expression by targeting its 3′-UTR, resulting in increased proliferation and migration of U87 and U251 cells *in vitro*, as well as tumor growth in the U87 mouse model	Li et al. (2015)
Glioma	U251, U87, and A172 cells	miR-134-5p; Circ-ZNF609	In glioma cells, miR-134-5p negatively regulates *BTG2* expression and inhibits cell proliferation and migration, while Circ-ZNF609 positively regulates *BTG2* expression through competitive binding to miR-134-5p and induces cell proliferation and migration	Tong et al. (2020)
Glioma (GBM)	• Glioma stem cells (GSCs) • GBM orthotopic xenografts derived from GSCs	*Piwil1*; *c-Myc*; *Olig2*; *Nestin*	In GSCs, *Piwil1* silencing significantly increases *BTG2* expression, leading to reduced *c-Myc*, *Olig2*, and *Nestin* expression, and thus cell cycle arrest and apoptosis; *in vivo*, this results in suppressed tumor growth and increased mouse survival	Huang et al. (2021)
Medulloblastoma	*Ptch1* ^+/−^/Tg*PC3* mice	*Cyclin D1*; *Math1*; *NeuroD1*; *NeuN*	Overexpression of *PC3*/*BTG2* in GCPs causes a reduction in cell proliferation, due to the inhibition of *cyclin D1* gene transcription, and an increase in cell differentiation, due to the increased expression of *Math1*, *NeuroD1* and *NeuN* genes, resulting in a 40% reduction in MB incidence	Farioli-Vecchioli et al. (2007)
Medulloblastoma	*Ptch1* ^+/−^/*Tis21* ^KO^ mice	*Cxcl3*	In *Ptch1* ^+/−^ mice, *Tis21*/*BTG2* deficiency causes a significant increase in the incidence of MB, due to the impaired migration of GCPs from the cerebellar surface to the inner layers during development: this phenotype is caused by the downregulation of the chemokine *Cxcl3*, whose transcription is directly controlled by Tis21/BTG2	Farioli-Vecchioli et al. (2012a)
Medulloblastoma	*Ptch1* ^+/−^/*Tis21* ^KO^ mice	*Cxcl3*	In 1-month-old *Ptch1* ^+/−^/*Tis21* ^KO^ mice, chronic administration (for 4 weeks) of the chemokine Cxcl3 by intracerebellar implantation of Alzet osmotic minipumps completely suppresses the development of MB lesions, forcing pGCPs to leave the lesions and differentiate	Ceccarelli et al. (2016)
Medulloblastoma	Athymic nude mice subcutaneously grafted with *Ptch1* ^+/−^ MB cells	*NeuroD1*; *NeuN*; *Cyclin D1*; *Cyclin E*; *cdc6*	Gene therapy with an adeno-associated virus carrying the *Tis21*/*BTG2* gene is able to slow the growth of MB allografts, significantly reducing their volume, through the inhibition of cell proliferation (downregulation of *cyclin D1*, *cyclin E* and *cdc6* genes) and the improvement of neural differentiation (upregulation of *NeuroD1* and *NeuN* genes)	Presutti et al. (2018)
Medulloblastoma	• GCPs, DAOY, MED341 • Shh-MB Neurod2-SmoA1 transgenic mice	*Gli2*; *FoxD1*; Nkx2-2as	In GCPs, the long noncoding RNA Nkx2-2as sequesters miR-103 and miR-107, derepressing their target *BTG2* and preventing cell division and migration; in the context of Shh-MB, Shh signaling impairs Nkx2-2as expression by upregulating the transcriptional repressor *FoxD1 via* Gli2, resulting in repression of the MB suppressor *BTG2*	Zhang et al. (2018)
Medulloblastoma	• *Ptch1^+/−^/Tis21^KO^* mice • Athymic nude mice subcutaneously grafted with *Ptch1^+/−^/Tis21^KO^* MB cells	PI3K/AKT/mTOR pathway • activators: *Pdgfd*, *Deptor*, *Dgkq*, *Rraga* • inhibitors: *Smg1* • components: AKT, S6, 4EBP1	In *Ptch1* ^+/−^/*Tis21* ^KO^ mice, activation of the PI3K/AKT/mTOR pathway in neoplastic GCPs causes a significant increase in proliferation and a significant decrease in apoptosis, paralleled by hyperphosphorylation of AKT and the mTOR target S6. This phenotype is reversed in nodules obtained by engrafting primary *Ptch1* ^+/−^/*Tis21* ^KO^ MBs into immunosuppressed mice by treatment with the PI3K inhibitor MEN1611, resulting in a significant reduction in tumor growth	Ceccarelli et al. (2021)
Medulloblastoma	• *Ptch1^+/−^/Tis21^KO^* mice • MB orthotopic xenografts derived from DAOY	*Cxcl3*	In 3-month-old *Ptch1* ^+/−^/*Tis21* ^KO^ mice, 4-week administration of the chemokine Cxcl3 is not sufficient to completely eradicate the tumor mass, but causes a seven-fold reduction in tumor volume by inducing the migration and differentiation of pGCPs from the lesions to the inner cerebellar layers. Furthermore, in immunosuppressed mice xenografted with the metastatic MB cell line DAOY, exogenous Cxcl3 administration does not significantly affect the frequency of metastases	Ceccarelli et al. (2025)
*BTG1* in malignant brain tumors				
Glioma	U87 and T98G cells	*Cyclin D1*; *p21*; *VEGF*; *cleaved caspase-3*; *Wnt1*; *β-catenin*	*BTG1* expression negatively correlates with cell viability, inducing cell cycle arrest and apoptosis, as well as with migration, invasion, and angiogenesis, by modulating the expression of *cyclin D1*, *p21*, *VEGF*, and *cleaved caspase-3* genes; furthermore, *BTG1* significantly regulates the expression of *Wnt1* and *β-catenin* genes, likely by controlling the activation of the Wnt/β-catenin pathway	Qian et al. (2019)
Glioma (GBM)	U87 and U251 cells	*PUM2*	*PUM2* positively regulates GBM cell proliferation, reduces apoptosis, and promotes cell migration and invasion by reducing *BTG1* expression by targeting its 3′-UTR	Wang et al. (2019)
Medulloblastoma	*Ptch1* ^+/−^/*BTG1* ^KO^ mice	*Cyclin D1*; *cleaved caspase-3*; *PRMT1*; *CD15*	In *Ptch1* ^+/−^ mice, genetic ablation of *BTG1* does not increase the frequency of MB, nor the proliferation of neoplastic GCPs, but significantly increases their apoptosis (*cleaved caspase-3* ^+^ cells), probably as a consequence of the increased expression of *PRMT1*; furthermore, genetic ablation of *BTG1* causes a significant increase in *CD15* ^+^ cancer stem cells	Ceccarelli et al. (2020a)

GBM: glioblastoma; GCP: granule cell precursor; GSC: glioma stem cell; MB: medulloblastoma; pGCP: preneoplastic GCP.

In 2008, Calzolari and colleagues observed in a murine glioma model they generated by overexpressing platelet-derived growth factor B (PDGF-B) that *BTG2* downregulation was involved in the acquisition of tumorigenic potential by PDGF-B-induced gliomas, i.e., in the progression of low- to high-grade gliomas capable of giving rise to secondary tumors following intracranial transplantation in adult mice (Calzolari et al., 2008). Indeed, they observed that *BTG2* was strongly downregulated in high-grade tumors compared to low-grade ones, which was in agreement with previous observations that *BTG2* deregulation cooperates with PDGF-B in promoting glioma formation (Johansson et al., 2004; Johansson Swartling, 2008). Confirmation of the role of *BTG2* in glioma progression came from a subsequent study by the same group. Appolloni et al. (2012) demonstrated that *BTG2* downregulation by itself is not sufficient to allow tumor progression, but is required to confer fully malignant characteristics to PDGF-B-induced glioma cells. Interestingly, this role of *BTG2* downregulation as a promoter, rather than an initiator, in tumor development was also observed in a mouse model of spontaneous medulloblastoma (see below). The authors observed that in the PDGF-B-induced glioma cells *BTG2* overexpression caused a ten-fold reduction in *cyclin D1* mRNA levels compared to untransduced cells, consistent with the known effect of BTG2 on *cyclin D1* expression (Guardavaccaro et al., 2000; Farioli-Vecchioli et al., 2007), without however modifying the fraction of proliferating (Ki67^+^) or apoptotic cells, suggesting that in glioma cells *BTG2* overexpression causes an increase in cell cycle length rather than cell cycle exit. The authors therefore concluded that the loss of *BTG2* expression was compatible with the survival of PDGF-B overexpressing cells and their progression to full-blown malignancy (Appolloni et al., 2012). Most importantly, Appolloni and colleagues conducted a meta-analysis of public datasets of human gliomas and demonstrated that patients with a *BTG2* expression level twice lower than the average had a significantly worse prognosis than those with a high level of *BTG2* expression (Appolloni et al., 2012). Overall, these findings suggested that the role of *BTG2* in glioma progression is not limited to PDGF-B-induced glioma but also extends to human tumors, and encouraged further analyses aimed at identifying the mechanisms underlying *BTG2* actions in glioma development.

Several groups have shown that in glioma cells *BTG2* is a target gene for some miRNAs. Li and colleagues demonstrated that miR-27a negatively regulated endogenous *BTG2* expression by targeting its 3′-UTR, resulting in increased G1-to-S transition and proliferation of GBM cells. Thus, miR-27a was able to modulate the clonogenic growth and migration of human U87 and U251 cells *in vitro*, as well as the growth of mouse xenografts bearing U87 cells, by directly targeting the *BTG2* gene (Li et al., 2015). Conflicting results were obtained by Tong et al. (2020). The authors found that miR-134-5p, which was downregulated in several GBM cell lines (U251, U87, and A172), acted as a tumor suppressor by inhibiting the proliferation and cell migration of glioma cells, as previously demonstrated (Zhong and Li, 2015; Zhao et al., 2016; Wang Z et al., 2018), and negatively regulated the expression of *BTG2*. In glioma cells, the authors observed that the circular RNA Circ-ZNF609 positively regulated the expression of *BTG2* through competitive binding to miR-134-5p, and acted as an oncogene by inducing the proliferation and migration of glioma cells (Tong et al., 2020). These findings, indicating that *BTG2* expression is oppositely regulated by miR-134-5p and CircZNF609 and positively correlates with tumor development, contradict previous findings and therefore deserve further investigation. Given the known antiproliferative and tumor suppressive nature of *BTG2*, we speculate that other players are likely involved in the CircZNF609/miR-134-5p/*BTG2* pathway that controls glioma proliferation and migration.

Despite aggressive therapy, GBM inevitably recurs, and patients succumb to the disease due to the rapid development of radio- and chemoresistance to anticancer drugs (Van Meir et al., 2010). Glioma stem cells (GSCs) are thought to be responsible for therapy resistance, and Huang et al. (2021) demonstrated the involvement of the *BTG2* gene in their biology (Huang et al., 2021). More specifically, the authors observed that Piwi-like protein 1 (Piwil1), an Argonaute protein that binds small RNAs, was preferentially expressed in perivascular GSCs and required for their survival and self-renewal, but not for migration and invasion. Notably, Piwil1 silencing markedly increased *BTG2* expression at both mRNA and protein levels, which in turn reduced the expression of *c-Myc* and the stem cell factors *Olig2* and *Nestin*. These global changes in GSC gene expression led to cell cycle arrest and apoptosis both *in vitro* and *in vivo*, where Piwil1 silencing — and subsequent *BTG2* overexpression — suppressed tumor growth and promoted survival in mice (Huang et al., 2021). Therefore, *BTG2* may represent a novel biomarker for GSCs and could potentially be manipulated to develop novel therapies for glioma.

As previously mentioned, *BTG1* was initially identified as a translocation partner of the *c-Myc* gene in B-cell lymphoblastic leukemia (Rimokh et al., 1991), and recent research has demonstrated its involvement in numerous solid tumors, as well as hematological malignancies (see above in this review; Yuniati et al., 2019; Ceccarelli et al., 2020a; Kim et al., 2022). In particular, low or absent levels of *BTG1* have been associated with uncontrolled tumor proliferation, high invasiveness, angiogenesis and metastasis, thus worse patient outcome, indicating *BTG1* as a tumor suppressor and diagnostic marker in various types of cancer (Zheng et al., 2022). See Table 4 and Figure 2.

In 2019, Qian and colleagues demonstrated that *BTG1* acts as a tumor suppressor also in glioma, preventing tumor development through repression of the Wnt/β-catenin pathway (Qian et al., 2019). First, the authors studied *BTG1* mRNA and protein levels in different GBM cell lines, finding that they were significantly lower than those in the normal glial HEB cell line. Next, the authors chose the U87 and T98G cell lines for their experiments, as the former had the lowest *BTG1* levels, while the latter had the highest *BTG1* expression levels. Therefore, using *in vitro* gene silencing or overexpression experiments, they demonstrated that *BTG1* expression was negatively correlated with glioma cell viability, inducing cell cycle arrest in the G0/G1 phase transition and apoptosis, glioma cell migration and invasion, and the angiogenic capacity of human umbilical vein endothelial cells. These effects were the result of differences in the expression levels of the cell cycle-related proteins *cyclin D1* and *p21*, *VEGF*, a promoter of blood vessel formation, and *cleaved caspase-3*, which plays a key role in apoptosis. Furthermore, in glioma cells, BTG1 significantly regulated the expression of the *Wnt1* and *β-catenin* genes, likely controlling the activation of the Wnt/β-catenin pathway, as suggested by *in vitro* experiments conducted with a Wnt/β-catenin pathway agonist (LiCl) and inhibitor (FH535) (Qian et al., 2019). These findings are consistent with the transcriptional co-regulatory activity of BTG1, which interacts with and recruits PRMT1 into transcription factor complexes (Lin et al., 1996; Berthet et al., 2002), modulating downstream gene expression: for example, the BTG1/PRMT1/ATF4 complex has been shown to control the survival/apoptosis balance in cells subjected to stress conditions, such as hypoxia and nutrient limitation, frequently observed in solid tumors (Yuniati et al., 2016). Furthermore, inhibition of *cyclin D1* expression by BTG1 has been observed in other brain cells such as cerebellar precursors (Ceccarelli et al., 2015).

In another 2019 paper, Wang and colleagues demonstrated that *BTG1* downregulation in GBM development may be a consequence of the action of the RNA-binding protein PUM2 (Wang et al., 2019). In the CNS, PUM2 is known to modulate neurogenesis by promoting neural stem cell proliferation through transcriptional repression of cell cycle genes (Zhang et al., 2017). The authors observed that *PUM2* mRNA and protein levels were higher in GBM tissues, as well as in several GBM cell lines, including U-87MG, U-T98G, U-251MG, and A172, compared to normal tissues. Through *PUM2* gene silencing experiments in U-251MG and U-87MG cells, Wang and colleagues demonstrated that PUM2 positively regulated GBM cell proliferation, reduced apoptosis, and promoted cell migration and invasion. These effects on GBM development were due to downregulation of *BTG1* expression: in fact, PUM2 directly bound to the 3′-UTR of *BTG1*, blocking mRNA translation and likely promoting its degradation, as previously demonstrated for other genes (Bermudez et al., 2011). Furthermore, *BTG1* knockdown was able to reverse the effect of *PUM2* knockdown on GBM cell proliferation, migration, and invasion (Wang et al., 2019). All these results, as well as those presented by Qian and colleagues (2019), were obtained only *in vitro* on GBM cell lines, which show low *BTG1* expression, as the authors did not conduct analyses of *BTG1* expression and mechanisms of action in patient samples.

Of note, the downregulation of *BTG1* expression in glioma could also be due to the negative regulatory action of eukaryotic initiation factor 3 (eIF3) subunits (Lee A et al., 2015), which have been strongly associated with glioma development (Chai et al., 2019; Krassnig et al., 2021). Alternatively, it could depend on the action of different miRNAs, which, initially identified in different tumor contexts (Li Y et al., 2014; Alanazi et al., 2015), are now known to be involved in the cellular processes underlying glioma development (Wang B et al., 2018; Wang H et al., 2018; Wei et al., 2019; Schnabel et al., 2021; Zhang et al., 2022).

Overall, these findings strongly support the hypothesis that *BTG1* acts as a tumor suppressor in glioma and may represent a promising therapeutic target for its treatment. However, it should be noted that two recent bioinformatics studies revealed that *BTG1* expression was higher in GBM tissue than in normal tissue (Bai et al., 2017; Zhang et al., 2023): therefore, the regulation of *BTG1* in brain tumors remains to be fully explored.

In summary, in the context of glioma, *BTG2* primarily controls cell cycle length, and therefore proliferation and survival, of tumor cells, including cancer stem cells, enabling tumor progression of low- to high-grade gliomas. Downregulation of *BTG2* in these tumors is due to translational inhibition by specific proteins, such as Piwil1, or miRNAs, such as miR-27a or miR-134-5p (Tables 4, 5), although the results obtained with the latter miRNA are unclear and contradict previous studies, thus requiring further investigation. Similar to *BTG2*, *BTG1* controls the cell cycle, and thus the proliferation and apoptosis, of glioma tumor cells, and its expression is negatively correlated with tumor cell migration and invasion. *BTG1*’s tumor suppressor function is exerted through the transcriptional control of cell cycle-related proteins and components of the Wnt/β-catenin pathway. In glioma cell lines, *BTG1* expression is negatively regulated by translational inhibitors such as miRNAs or the RNA-binding protein PUM2 (Tables 4, 5), unlike the high *BTG1* expression found in patient tumors: this highlights the need for further analysis to clarify the actual role of *BTG1* in human gliomas.

**TABLE 5:** Comparison of *BTG2* and *BTG1* in malignant brain tumors.

Tumor	Gene	Expression status	Main functional effects	Clinical/prognostic relevance
Glioma	*BTG2*	The expression level of *BTG2* in human gliomas is variable and correlates with the prognosis of patients	Overexpression of *BTG2* • In tumor cells, it causes an increase in cell cycle length • In GSCs, it leads to cell cycle arrest and apoptosisDownregulation of BTG2 • Increases tumor cell proliferation • Promotes cell migration • Induces the progression of low- to high-grade gliomas	• Downregulation of *BTG2* promotes glioma progression, thus *BTG2* may be considered a promising prognostic marker to predict disease progression • *BTG2* could represent a novel biomarker for GSCs and potentially be manipulated to develop new therapies for glioma
	*BTG1*	Bioinformatics studies indicate that *BTG1* expression is higher in glioma tissue than in normal tissue (unlike glioma cell lines)	Overexpression of *BTG1* • Reduces tumor cell proliferation • Increases tumor cell apoptosis • Inhibits cell migration and invasionDownregulation of *BTG1* • Increases tumor cell proliferation • Reduces tumor cell apoptosis • Promotes cell migration and invasion	• *BTG1* acts as a tumor suppressor in glioma and may represent a promising prognostic and therapeutic marker • Translational repressors acting upstream of *BTG1*, such as miRNAs or PUM2, or downstream targets of BTG1, such as the Wnt/β-catenin pathway, could be used to develop novel therapies for glioma
Medulloblastoma	*BTG2*	A broad deregulation of *BTG2* expression is observed in human MBs compared to control cerebellar samples	Overexpression of *PC3/BTG2* • Reduces the frequency of *MB* • Reduces cell proliferation • Increases cell differentiationGenetic ablation of *Tis21/BTG2* • Increases the incidence of MB • Impairs GCP migration by downregulating *Cxcl3* expression • Synergizes with the PI3K/AKT/mTOR pathway, with pro-proliferative and anti-apoptotic effects	• *BTG2* is a MB suppressor gene and a potentially relevant target for gene therapy • Downstream targets of BTG2, such as *Cxcl3*, could be successfully used alone or in combination with other targeted therapies (e.g., PI3K/AKT/mTOR pathway inhibitors) as therapeutic agents in human MB therapy
	*BTG1*	Some human MBs show extensive deregulation of *BTG1* expression, otherwise unchanged compared to control cerebellar samples	Genetic ablation of *BTG1* • Does not increase the frequency of MB • Does not affect tumor cell proliferation • Increases tumor cell apoptosis • Causes a significant increase in CSCs	• *BTG1* regulates the balance between tumor cell proliferation and survival • *BTG1* may be implicated in MB recurrence as it influences CSC biology

CSC: cancer stem cell; GCP: granule cell progenitor; GSC: glioma stem cell.

### 7.2 Medulloblastoma

Medulloblastoma (MB) is a malignant, highly invasive primitive neuroectodermal tumor that typically arises in the cerebellum, in the posterior fossa of the skull. The disease is often associated with increased intracranial pressure; the predominant symptoms are headache, chronic nausea, vomiting, and ataxia (Crawford et al., 2007; Northcott et al., 2019). MB cells disseminate early within the CNS: approximately one-third of patients have metastases in the cerebrospinal fluid and meninges at diagnosis. More rarely, MB spreads to extraneural sites such as bone, bone marrow, lymph nodes, liver, and lungs (MacDonald et al., 2003; Van Ommeren et al., 2020). Most cases are sporadic, though hereditary syndromes (Gorlin, Li-Fraumeni, Turcot) may predispose to MB (Foulkes et al., 2017; Waszak et al., 2018; Kolodziejczak et al., 2023).

Although MB can occur at any age, it is predominantly pediatric and represent the most common childhood malignant brain tumor, accounting for approximately 20% of primary CNS tumor in patients under 19 years of age (Crawford et al., 2007; Ostrom et al., 2018). In adults, MB accounts for 0.4%–1% of brain tumors (Smoll and Drummond, 2012; Franceschi et al., 2022).

Historically, MB patients were stratified into two groups, standard risk and high risk, based on the patient’s age, the presence of metastases at diagnosis, and the size of residual tumor after surgery. Standard risk patients are older than 3 years, have no metastases, and have a residual tumor mass of <1.5 cm^2^ after surgery (Martin et al., 2014). Current multimodal therapy for MB includes maximal safe resection, high-dose chemotherapy, and, in non-infant patients, craniospinal irradiation, and allows 5-year disease-free survival rates of 80% for patients with standard-risk MB and approximately 60% for high-risk disease (Martin et al., 2014; Ramaswamy et al., 2016; Bailey et al., 2022; He D et al., 2024). However, approximately 30% of patients relapse, and survival after relapse is less than 5% (Hill et al., 2021). Furthermore, long-term survivors suffer severe adverse effects, such as physical and neurocognitive deficits (Edelstein et al., 2011; Fay-McClymont et al., 2017) and secondary malignancies (Packer et al., 2013; Nantavithya et al., 2020). New and more effective therapeutic strategies are therefore urgently needed to reduce treatment-related toxicities, significantly improving the quality of life of young patients.

Over the past 2 decades, molecular profiling has transformed the prognosis, risk stratification, and management of childhood MB, opening the possibility of precision medicine approaches. Based on the molecular profiling, MBs can be classified into four main subgroups of clinical relevance (Wnt, Sonic hedgehog (Shh), group 3 and group 4), with further subclassification possible within each subgroup (Ramaswamy et al., 2016; Juraschka and Taylor, 2019; Sidaway, 2021; Sursal et al., 2022).

The Wnt subgroup accounts for approximately 10% of human MBs, which harbor activating mutations in components of the Wnt signaling pathway (Juraschka and Taylor, 2019; Northcott et al., 2019). This subgroup has the best prognosis, probably due to alterations in the patients’ blood-brain barrier, which is therefore more permeable to systemic chemotherapy (Phoenix et al., 2016). Furthermore, Wnt tumors rarely metastasize or recur (Juraschka and Taylor, 2019): therefore, Wnt patients are considered low risk (Ramaswamy et al., 2016). It has been suggested that the cellular origin of this subgroup is located within the lower rhombic lip and dorsal brainstem (Gibson et al., 2010; Grammel et al., 2012).

Group 3 and group 4 MBs show the worst survival outcomes, are often metastatic at diagnosis, and lack targeted therapies, as their molecular pathogenesis is not yet well understood. Group 3 represents approximately 25% of all MBs and is proposed to originate from neural stem cells. Group 4 represents 35%–40% of all MB diagnoses, and the putative cells of origin for these tumors may reside in the upper rhombic lip compartment (Juraschka and Taylor, 2019; Vladoiu et al., 2019; Lin et al., 2016).

The Shh subgroup comprises approximately one-third of all human MBs, with a variable outcome based on the clinical and molecular characteristics of the tumors (Juraschka and Taylor, 2019; Sidaway, 2021). Interestingly, to this group belongs the large majority of published MB animal models (Wu et al., 2011; Goodrich et al., 1997; Lee et al., 2007; Hatton et al., 2008; Grigore et al., 2023). Shh-dependent MBs arise from cerebellar granule precursor cells (GCPs), which harbor activating mutations or copy number alterations in components of the Shh signaling pathway, which controls their proliferation during the physiological cerebellar development (Juraschka and Taylor, 2019; Northcott et al., 2019). In humans, cerebellar development begins in the first trimester of gestation and persists for several months after birth; in mice, this process begins during early embryonic stages and continues up to 3 weeks after birth. During cerebellar development, GCPs migrate from the upper rhombic lip to the surface of the embryonic cerebellar anlage, where they form the external granular layer (EGL). Postnatally, within this layer, GCPs undergo extensive proliferation and expansion in response to the mitogen Shh, secreted by underlying Purkinje neurons. Subsequently, GCPs exit the cell cycle and migrate inward — past the molecular (ML) and Purkinje (PL) layers — toward the internal granular layer (IGL), where they ultimately differentiate into mature granule neurons (Dahmane and Ruiz i Altaba, 1999; Wechsler-Reya and Scott, 1999; Wallace, 1999; Wang and Zoghbi, 2001). Proper cerebellar patterning depends on tightly regulated programs of proliferation, differentiation, migration, and survival of glial and neuronal cells; perturbations underlie developmental disorders and cancer (Ceccarelli, 2023).

It is known that several genes important for cell cycle control can act, if their expression is altered, as Shh-MB drivers (Skowron et al., 2021). Deregulation of *BTG2* expression has been observed in human MB tumors (Farioli-Vecchioli et al., 2007; Ceccarelli et al., 2021), as well as some MB samples showed a large deregulation (up to 40%) of *BTG1* expression (Ceccarelli et al., 2020a).

As previously mentioned, *PC3*/*Tis21*/*BTG2* is expressed, albeit with different spread and intensity, in the layers (EGL, ML, PL, IGL) of the cerebellar cortex, where it inhibits cell proliferation and induces neuronal maturation during the embryonic and early postnatal period. In mice, *PC3*/*BTG2* overexpression has been observed to cause a marked decrease in cell proliferation in GCPs within the EGL, following the inhibition of *cyclin D1* expression, and an increase in the expression of *Math1*, a transcription factor required for GCP maturation. Furthermore, *PC3*/*BTG2* overexpression has been shown to cause increased cellular differentiation throughout the cerebellum, with a significant increase in ML and IGL thickness and the number of NeuroD1-positive cells in the EGL. Overall, in mice, *PC3*/*BTG2* overexpression has been shown to increase the production of postmitotic neurons, resulting in reduced cerebellar development, abnormal posture, and ataxic gait (Canzoniere et al., 2004).

The antiproliferative and prodifferentiative effects of *PC3*/*BTG2* in GCPs suggested a possible role of this gene as a tumor suppressor of Shh-induced MBs. See Table 4 and Figure 1. This hypothesis was tested by Farioli-Vecchioli and colleagues in a mouse model obtained by crossing transgenic mice conditionally expressing the *PC3*/*BTG2* gene (Tg*PC3*) in the cerebellar GCPs with *Patched1* heterozygous mice (*Ptch1* ^+/−^) (Farioli-Vecchioli et al., 2007). *Ptch1* ^+/−^ mice are spontaneously predisposed to develop MB due to the constitutive activation of the Shh pathway, caused by the lack of the Ptch1 receptor that inhibits the pathway (Goodrich et al., 1997; Hahn et al., 1998). Additionally, more than 50% of *Ptch1* ^+/−^ mice present preneoplastic nodular formations containing highly proliferating preneoplastic GCPs (pGCPs) on the surface of the cerebellum and in the interlobular fissures within 2–6 months of birth, representing the initial stages of MB development (Goodrich et al., 1997; Oliver et al., 2005; Kessler et al., 2009).

In *Ptch1* ^+/−^/Tg*PC3* mice, *PC3*/*BTG2* overexpression caused an approximately 40% decrease in the MB incidence, as well as a marked reduction in the number of preneoplastic lesions. In the cerebellum of these mice, the authors observed a reduction in cell proliferation, due to the negative control of *PC3*/*BTG2* on the transcription of the *cyclin D1* gene, and an increase in cell differentiation, due to increased expression levels of the neurogenic transcription factors *NeuroD1* and *Math1,* accompanied by expression of the mature neuron marker *NeuN* (Farioli-Vecchioli et al., 2007).

Similar results were observed when MB allografts, obtained by subcutaneous engraftment of *Ptch1* ^+/−^ MB cells in athymic nude mice, were injected with an adeno-associated virus carrying the *Tis21*/*BTG2* gene. Presutti et al. (2018) demonstrated that treatment with this adenovirus significantly reduced the volume of tumor nodules compared to treatment with an empty adenovirus used as a control. Gene therapy with *Tis21*/*BTG2* was able to slow the growth of MB tumors through significant inhibition of cell proliferation and enhancement of neural differentiation (Presutti et al., 2018). These results validated *BTG2* as a relevant target for MB therapy.

Consistently, *Tis21*/*BTG2* deletion has been shown to increase the MB incidence in *Ptch1* heterozygous mice crossed with *Tis21* knockout mice (Farioli-Vecchioli et al., 2012a). *Ptch1* ^+/−^ mice develop spontaneous MBs within the first year of life with a low incidence depending on the background (Wetmore et al., 2000; Pazzaglia et al., 2002). The incomplete penetrance of tumor formation in these mice suggests that additional signaling pathways cooperate with Shh signaling to promote MB development (Rao et al., 2004). In *Ptch1* ^+/−^/*Tis21* ^KO^ mice, *Tis21*/*BTG2* deficiency resulted in a significant increase of MB incidence compared to the incidence of spontaneous tumors in *Ptch1* ^+/−^ mice (80% vs. 25.3%), with a higher frequency and persistence of preneoplastic lesions. Surprisingly, this phenotype was not due to increased GCP proliferation, suggesting that other genes belonging to the same family, such as *BTG1*, could substitute for the known antiproliferative action of *Tis21*/*BTG2* in the cerebellum (see below). Rather, the *Ptch1* ^+/−^/*Tis21* ^KO^ phenotype was due to impaired GCP migration from the cerebellar surface to the inner layers during cerebellar development, accompanied by a defect in cell differentiation (Farioli-Vecchioli et al., 2012a). Indeed, the permanence of GCPs in the proliferative niche of the EGL under the control of Shh exponentially increases the possibility of neoplastic transformation, demonstrating the crucial role of cerebellar GCP migration in MB tumorigenesis (Farioli-Vecchioli et al., 2012a; 2013; Haag et al., 2012). Of note, the migration defect of GCPs, specifically dependent on *Tis21*/*BTG2* deficiency, causes cell persistence in the EGL, which, however, alone is not sufficient for tumorigenesis. Only in *Ptch1* ^+/−^ mice, which are spontaneously predisposed to the development of MB, can the migration defect trap GCPs in an environment that facilitates their continued growth and survival. Therefore, similar to what has been described in the context of glioma, *BTG2* downregulation acts as a tumor promoter, rather than an initiator, in the development of MB. By genome-wide analysis, Farioli-Vecchioli and colleagues found that downregulation of the chemokine *Cxcl3* was responsible for the defective migration of normal and preneoplastic GCPs in *Ptch1* ^+/−^/*Tis21* ^KO^ mice. The authors observed that *Cxcl3* gene transcription was directly controlled by *Tis21*/*BTG2*, as is also known for *cyclin D1*, *Id3*, and *RAR-β* (Passeri et al., 2006; Farioli-Vecchioli et al., 2007; 2009), and that Cxcl3 was able to regulate GCP migration in a cell-autonomous manner without affecting either their proliferation or differentiation (Farioli-Vecchioli et al., 2012a). These results suggested that the chemokine Cxcl3, which plays a key role in GCP migration and MB onset, may represent a novel therapeutic target for MB.

Since pGCPs within preneoplastic lesions can still undergo migration and differentiation like normal GCPs, although they are capable of generating tumors when transplanted (Yang et al., 2008; Kessler et al., 2009), Ceccarelli and colleagues tested whether the migration-promoting action of Cxcl3 could induce pGCPs to migrate out of MB lesions and differentiate, thereby reducing tumor formation. To this end, the authors subjected *Ptch1* ^+/−^/*Tis21* ^KO^ mice to chronic administration of the chemokine Cxcl3 by intracerebellar implantation of Alzet osmotic minipumps. First, the authors started Cxcl3 treatment in *Ptch1* ^+/−^/*Tis21* ^KO^ mice 1 month after birth, a time when lesions have already started to develop and pGCPs exhibit the greatest intrinsic plasticity (Kessler et al., 2009): the authors demonstrated that intracerebellar infusion of exogenous Cxcl3 for 4 weeks completely suppresses the development of MB lesions, forcing pGCPs to leave the lesions and differentiate (Ceccarelli et al., 2016). Subsequently, the same group extended their previous analyses by subjecting 3-month-old *Ptch1* ^+/−^/*Tis21* ^KO^ mice — bearing irreversibly tumor-committed MB lesion — to intracerebellar infusion of Cxcl3, to test whether the chemokine has a migration-promoting action on pGCPs even in the advanced stages of MB tumorigenesis. The authors demonstrated that a 4-week treatment with Cxcl3 was not sufficient to completely eradicate the tumor mass, but caused a seven-fold reduction in tumor volumes, inducing pGCP migration and differentiation from the lesions to the inner cerebellar layers (Ceccarelli et al., 2025). Furthermore, the authors tested whether the pro-migratory action of Cxcl3 could increase the risk of metastatic spread. Since the *Ptch1* ^+/−^ model develops localized MBs, which do not disseminate into the cerebrospinal fluid and do not generate leptomeningeal and spinal cord metastases (Wu et al., 2012), Ceccarelli and colleagues generated an orthotopic MB model by xenotransplanting a metastatic MB cell line into the cerebellum of immunosuppressed mice. In this model, the authors observed that the administration of exogenous Cxcl3 did not significantly influence the frequency of metastases: this result, and the fact that Cxcr2 – the Cxcl3 receptor – was variably expressed in all MB subgroups (Ceccarelli et al., 2025), suggested that Cxcl3 could be successfully used for human MB therapy. Further studies will be needed to verify whether the antitumor effect of Cxcl3 observed in preclinical models also occurs in patients. Orthotopic xenograft models are likely not ideal for studying the response to Cxcl3 treatment, as in them, exogenously transplanted human tumor cells come into contact with surrounding healthy tissue, in the absence of a tumor microenvironment (TME). Indeed, the TME is a dynamic niche composed of blood vessels, functional proteins, and non-tumor cells, such as fibroblasts, immune cells, and endothelial cells, which interacts with tumor cells to promote immune evasion, survival, and recurrence (Zhou et al., 2026).

In addition to Cxcl3, whole-genome analysis of *Ptch1* ^+/−^/*Tis21* ^KO^ mice has allowed the identification of 187 other gene sequences – 163 of which have a functional product – whose expression significantly differs with respect to *Ptch1* ^+/−^ mice (Farioli-Vecchioli et al., 2012a; Gentile et al., 2016). Among these genes, several components of the PI3K/AKT/mTOR pathway were found. In *Ptch1* ^+/−^/*Tis21* ^KO^ mice, *Tis21*/*BTG2* deletion was found to act synergistically with the *Ptch1* ^+/−^ genotype to activate the PI3K/AKT/mTOR pathway, as the expression levels of some pathway inhibitors (e.g., *Smg1*) were downregulated and several pathway activators (e.g., *Pdgfd*, *Deptor*, *Dgkq*, *Rraga*) were upregulated (Ceccarelli et al., 2021). Ceccarelli and colleagues demonstrated that in *Ptch1* ^+/−^/*Tis21* ^KO^ mice, activation of the PI3K/AKT/mTOR pathway caused a significant increase in proliferation and a significant decrease in apoptosis of neoplastic GCPs, paralleled by hyperphosphorylation of AKT and the mTOR target S6. This phenotype was reversed in nodules obtained by engrafting primary *Ptch1* ^+/−^/*Tis21* ^KO^ MBs into immunosuppressed mice by treatment with MEN1611, a PI3K inhibitor, resulting in a significant reduction in tumor growth. Overall, these data confirmed that PI3K/AKT/mTOR pathway activation contributes, together with the Cxcl3-dependent migration defect, to induce GCP transformation and, therefore, high-frequency tumorigenesis in *Ptch1* ^+/−^ mice lacking the *Tis21*/*BTG2* gene (Ceccarelli et al., 2021).

The role of *BTG2* as a MB suppressor and the existence of a cross-talk between *BTG2* and the Shh pathway in the pathogenesis of Shh-type MB were further validated by the work of Zhang and colleagues in 2018. The authors demonstrated, both *in vitro* and in spontaneous Shh-MB *Neurod2*-*SmoA1* transgenic mice, a mouse model with a higher incidence of tumors than the *Ptch1* ^+/−^ model (Wu et al., 2011), the existence of a Gli2/FoxD1/Nkx2-2as/*BTG2* axis, which is critically involved in the development of Shh-induced MB. The authors observed that in cerebellar cells, the long noncoding RNA Nkx2-2as functioned as a competitive endogenous RNA (ceRNA) to sequester miR-103 and miR-107, thereby derepressing their target *BTG2* and preventing cell division and migration. In the context of Shh-MB, Shh signaling impaired Nkx2-2as expression by upregulating the transcriptional repressor *FoxD1 via* Gli2, resulting in repression of the MB suppressor *BTG2* (Zhang et al., 2018). Thus, the authors provided new insights into the mechanisms underlying Shh-induced MB pathogenesis and confirmed *BTG2* as a candidate target for clinical MB treatment.

Similar to *BTG2*, *BTG1* is also highly expressed in cerebellar precursors and mature granule neurons during development and adulthood, respectively (Farioli-Vecchioli et al., 2012b; 2014b). *BTG1* plays a key role in cerebellar maturation and function, primarily regulating GCP proliferation through inhibition of *cyclin D1* (Ceccarelli et al., 2015). See Table 4 and Figure 2. Ceccarelli and colleagues observed that during cerebellar development, *BTG1* deletion caused a significant increase in GCP proliferation in the EGL, with mild but significant secondary deficits in differentiation and migration — the latter process being essentially controlled by *Tis21*/*BTG2*. Thus, in the cerebella of *BTG1* ^KO^ mice, a significant increase in EGL thickness was observed. The gradual disappearance of this layer during cerebellar maturation occurred due to the significant increase in apoptosis of *BTG1*-null GCPs, but on a longer time scale than in wild-type cerebella. Consequently, in *BTG1* ^KO^ mice, a permanent increase in adult cerebellar volume and an impairment of cerebellar-dependent motor coordination were observed (Ceccarelli et al., 2015).

The hyperproliferation of GCPs and increased EGL thickness observed in *BTG1* ^KO^ mice suggested that *BTG1* may be involved in the pathogenesis of Shh-MB by acting as a tumor suppressor. This hypothesis was tested by Ceccarelli et al. (2020a) in a MB mouse model (*Ptch1* ^+/−^/*BTG1* ^KO^) obtained by crossing *Ptch1* ^+/−^ mice with mice lacking *BTG1* gene. In *Ptch1* ^+/−^/*BTG1* ^KO^ mice, genetic ablation of *BTG1* did not increase the frequency of MB, either in preneoplastic stages or in established tumors. Surprisingly, it did not affect the proliferation of neoplastic GCPs, but significantly increased their apoptosis, likely as a consequence of increased expression of BTG1’s partner *PRMT1*, which targets apoptotic genes (Ceccarelli et al., 2020a). These results indicated that *BTG1* plays a role in MB pathogenesis by regulating the balance between tumor cell proliferation and survival; at the same time, they did not rule out its possible role as a MB suppressor, given that the pronounced increase in apoptosis following *BTG1* ablation could hide any increase in proliferation and tumorigenesis. Interestingly, in *Ptch1* ^+/−^/*BTG1* ^KO^ MBs the authors observed a significant increase in tumor cells expressing the carbohydrate antigen CD15 (Ceccarelli et al., 2020a), a surface marker of MB cancer stem cells (CSCs) (Read et al., 2009; Ward et al., 2009). This finding was in line with the fact that in adult neurogenic niches, *BTG1* is required for the physiological maintenance of stem/progenitor cell quiescence and self-renewal (see above). CSCs are a subpopulation of tumor cells within MBs that, by resisting chemotherapeutic agents and radiotherapy, are responsible for cancer recurrence and metastatic dissemination. Indeed, CSCs are resistant to common therapeutic approaches because they have a high capacity to repair DNA damage and export chemotherapeutic drugs out (Atashzar et al., 2020). Most importantly, CSCs are slow-proliferating/quiescent cells, which enter the cell cycle only occasionally, thus escaping antiproliferative therapeutic strategies (Aguirre-Ghiso, 2007). Thus, the *BTG1* gene was found to be involved in CSC proliferation and survival and may be implicated in MB recurrence and long-term treatment resistance.

In summary, during postnatal cerebellar development, *BTG1* and *BTG2* play different key roles in GCP biology: the former specifically controls their proliferation through the inhibition of *cyclin D1,* while the latter is essentially required for their migration through the transcriptional control of the chemokine *Cxcl3*. This translates into different roles in cerebellar tumorigenesis. In the context of Shh-induced MBs, *BTG2* acts as a tumor suppressor, as its overexpression reduces MB frequency by inhibiting cell proliferation and inducing cell differentiation, while its deletion increases tumor frequency due to impaired GCP migration (Tables 4, 5). In contrast, *BTG1* does not appear to be a canonical tumor suppressor, as its deletion does not affect tumor cell proliferation and does not increase MB frequency. However, its role in MB pathogenesis cannot be ruled out, given its involvement in CSC proliferation and survival (Tables 4, 5). Future studies are therefore needed to evaluate whether the *BTG1* gene is implicated in MB recurrence.

### 7.3 Therapeutic implications

As mentioned above, *PC3*/*Tis21*/*BTG2* and *BTG1* are expressed in the developing and adult CNS, where they control the biology of neural and glial cells. Furthermore, they are known tumor suppressors in various types of solid and hematological tumors. Therefore, as described, several groups have studied the action of *PC3*/*Tis21*/*BTG2* and *BTG1* on malignant brain tumors to identify novel molecular targets for use as diagnostic and prognostic markers in cancer patients and to develop alternative therapeutic approaches to conventional treatment regimens.

In gliomas, *BTG2* has been shown to control tumor progression, as its downregulation confers fully malignant characteristics to low-grade gliomas (Appolloni et al., 2012) (Table 4). Furthermore, downregulation of this gene correlates with the survival and self-renewal of cancer stem cells (Huang et al., 2021) (Table 4). Therefore, *BTG2* is a promising prognostic marker for predicting disease progression, and its expression level, which varies from patient to patient, correlates with prognosis (Table 5). In contrast, *BTG1* expression is not predictive, as it appears higher in glioma tissue than in normal tissue (Bai et al., 2017; Zhang et al., 2023). Since this gene acts as a tumor suppressor *in vitro* in glioma cells (Table 4), *BTG1* and its upstream/downstream targets might be better suited as direct targets, rather than biomarkers, for the development of novel glioma therapies (Table 5).

In the context of Shh-MB, *Tis21*/*BTG2* has proven to be a potentially relevant therapeutic target, as its overexpression *via* gene therapy has been shown to be therapeutically effective in preclinical models (Presutti et al., 2018) (Table 4). Similarly, exogenous administration of its transcriptional target, the chemokine Cxcl3, which mimics *BTG2*-controlled morphogenetic migration of GCPs, in a preclinical model of *Tis21*/*BTG2*-deficient MB has been shown to be effective in counteracting tumor development in both early and advanced stages, without risk of toxicity or metastasis (Ceccarelli et al., 2016; 2025) (Table 4). Therefore, *BTG2* and its downstream effector *Cxcl3* emerge as compelling therapeutic candidates for MB, either as stand-alone interventions or in combination with other targeted approaches, such as inhibitors of the PI3K/AKT/mTOR or Shh pathways or CAR-T cells (Table 5). As a diagnostic and prognostic marker, *BTG2* is not very reliable, as its expression is widely dysregulated in human MB (Table 5): an exception is represented by a small portion of patients with simultaneous *BTG2* downregulation and PI3K/AKT/mTOR pathway activation, who are classified as high-risk Shh-type MB with a very poor prognosis (Ceccarelli et al., 2021). *BTG1* expression is also widely dysregulated — often due to mutations — in human MB (Ceccarelli et al., 2020a), making this gene unsuitable as a biomarker for patient stratification. More interesting is its use as a therapeutic target, as manipulating *BTG1* expression could alter the balance between proliferation and apoptosis in MB tumor formation, with antitumor effects (Table 4, 5). However, in a preclinical mouse model, downregulation of *BTG1* has been shown to cause a marked increase in cancer stem cells (Ceccarelli et al., 2020a), known to be responsible for tumor recurrence over time and resistance to therapies. Further studies will therefore be needed to evaluate the risk/benefit ratio of manipulating the *BTG1* pathway in MB, as well as the *BTG2* pathway in glioma, given the role of these genes in cancer stem cell biology.

## 8 Conclusion

*PC3*/*Tis21*/*BTG2* and *BTG1* emerge as pivotal regulators of neural development and brain tumorigenesis, acting primarily through their ability to modulate cell cycle progression, differentiation, and apoptosis. In particular, *PC3*/*Tis21*/*BTG2* is essential for the differentiation of progenitor cells in adult neurogenic niches such as the hippocampus and SVZ, whereas *BTG1* plays a key role in maintaining stem cell quiescence and preserving the neural stem cell pool.

Their tumor-suppressive functions are tightly linked to the control of neural stem and progenitor cell dynamics, which, when disrupted, contribute to the onset and progression of aggressive brain tumors such as glioma and MB.

By promoting neuronal differentiation and migration, *PC3*/*Tis21*/*BTG2* reduces the susceptibility of cerebellar precursors to oncogenic transformation, while *BTG1* limits uncontrolled proliferation, including that of cancer stem cells implicated in tumor relapse.

To outline the key areas where evidence is lacking and suggest specific directions for future research, we should mention: i) the gene domain-specific causality *in vivo*; ii) the deadenylation specificity; iii) the apoptosis decision map, i.e., the rules underlying the anti-apoptotic and the pro-apoptotic activity of *BTG2* and *BTG1*; iv) the *BTG1*’s role in MB, in particular concerning cancer stem cells. Moreover, the Cxcl3 proof-of-concept is strong, but possible future directions at preclinical level are its testing in combination with CAR-T models or in combination with PI3K and Shh inhibitors.

The ability of these genes to regulate key molecular pathways underscores their potential as therapeutic targets and offers a promising avenue for personalized treatments. Future research should focus on strategies that modulate these cofactors — or their downstream effectors — to generate new therapeutic approaches that leverage their dual role in neurogenesis and tumor suppression.
